# Sensing chemical-induced DNA damage using CRISPR/Cas9-mediated gene-deletion yeast-reporter strains

**DOI:** 10.1007/s00253-024-13020-w

**Published:** 2024-02-01

**Authors:** Kosuke Yamamoto, Shintaro Tochikawa, Yuuki Miura, Shogo Matsunobu, Yuu Hirose, Toshihiko Eki

**Affiliations:** 1 Molecular Genetics Laboratory, Department of Applied Chemistry and Life Science, Toyohashi University of Technology, 1-1 Hibarigaoka, Tempaku-cho, Toyohashi, Aichi 441-8580 Japan; 2 Laboratory of Genomics and Photobiology, Department of Applied Chemistry and Life Science, Toyohashi University of Technology, 1-1 Hibarigaoka, Tempaku-cho, Toyohashi, Aichi 441-8580 Japan

**Keywords:** Yeast-based reporter assay, Luciferase, DNA crosslinker, DNA repair genes, Cell permeability

## Abstract

**Abstract:**

Microorganism-based genotoxicity assessments are vital for evaluating potential chemical-induced DNA damage. In this study, we developed both chromosomally integrated and single-copy plasmid–based reporter assays in budding yeast using a *RNR3* promoter–driven luciferase gene. These assays were designed to compare the response to genotoxic chemicals with a pre-established multicopy plasmid–based assay. Despite exhibiting the lowest luciferase activity, the chromosomally integrated reporter assay showed the highest fold induction (i.e., the ratio of luciferase activity in the presence and absence of the chemical) compared with the established plasmid-based assay. Using CRISPR/Cas9 technology, we generated mutants with single- or double-gene deletions, affecting major DNA repair pathways or cell permeability. This enabled us to evaluate reporter gene responses to genotoxicants in a single-copy plasmid–based assay. Elevated background activities were observed in several mutants, such as *mag1Δ* cells, even without exposure to chemicals. However, substantial luciferase induction was detected in single-deletion mutants following exposure to specific chemicals, including *mag1Δ*, *mms2Δ*, and *rad59Δ* cells treated with methyl methanesulfonate; *rad59Δ* cells exposed to camptothecin; and *mms2Δ* and *rad10Δ* cells treated with mitomycin C (MMC) and cisplatin (CDDP). Notably, *mms2Δ*/*rad10Δ* cells treated with MMC or CDDP exhibited significantly enhanced luciferase induction compared with the parent single-deletion mutants, suggesting that postreplication and for nucleotide excision repair processes predominantly contribute to repairing DNA crosslinks. Overall, our findings demonstrate the utility of yeast-based reporter assays employing strains with multiple-deletion mutations in DNA repair genes. These assays serve as valuable tools for investigating DNA repair mechanisms and assessing chemical-induced DNA damage.

**Key points:**

• *Responses to genotoxic chemicals were investigated in three types of reporter yeast.*

• *Yeast strains with single- and double-deletions of DNA repair genes were tested.*

• *Two DNA repair pathways predominantly contributed to DNA crosslink repair in yeast.*

**Supplementary Information:**

The online version contains supplementary material available at 10.1007/s00253-024-13020-w.

## Introduction

Restoring mutagen-induced DNA damage through DNA repair processes is crucial for maintaining cellular functions and genome integrity. DNA damage can lead to cell death or an increased risk of cancers due to accumulated genetic mutations (Friedberg et al. 2005). To assess the genotoxic potential of synthetic chemicals, various microorganism-based tests for detecting mutagens have been developed as alternatives to animal tests. For instance, the bacteria-based Ames test (Ames et al. 1973) is a widely used genotoxicity assay, despite some Ames-negative compounds demonstrating carcinogenicity in animals. In eukaryotes, yeast-based genotoxicity tests, using the budding yeast *Saccharomyces cerevisiae*, have been developed as supplements to animal- and cell-based assays (Eki 2018). Similar to bacteria-based tests, these assessments employ DNA alteration assays or reporter assays. *Saccharomyces cerevisiae*–based reporter assays use genes encoding enzymes or fluorescent proteins linked to DNA damage–responsive promoters, such as *RNR3* (Boronat and Pina 2006; Ichikawa and Eki 2006; Jia et al. 2002; Ochi et al. 2011; Wei et al. 2013), *RAD54* (Afanassiev et al. 2000; Boronat and Pina 2006; Walmsley et al. 1997), and *RAD51* (Liu et al. 2008). Transcriptional induction of these genes is triggered by genotoxic agents activating the DNA damage checkpoint pathway (Elledge et al. 1993). Given that yeast responds to DNA-damaging agents, similar to mammalian cells, yeast-based reporter assays are suitable for assessing potential genotoxicity in mammals.

Several cell-based bioassays incorporating yeast cells have been developed to screen potentially genotoxic chemicals. To enhance the sensitivity of yeast-based reporter assays, which is crucial for detecting low levels of mutagens, studies have focused on disrupting DNA repair functions and improving cell permeability to chemicals. Using DNA repair–deficient and/or cell wall–permeabilized yeast strains as hosts has successfully increased reporter gene product levels (Jia and Xiao 2003; Lichtenberg-Fraté et al. 2003; Walsh et al. 2005; Zhang et al. 2010, 2011). Previously, we developed a yeast-based assay system using sensor and β-galactosidase reporter plasmids, demonstrating increased sensitivity to genotoxic agents compared with the Ames test and conventional reporter systems (Ichikawa and Eki 2006). We also observed elevated reporter levels in yeast-based assays using the *Cypridina noctiluca* secretory luciferase gene and the *GFP* gene linked to the *RNR3* promoter (^P^*RNR3*) by using DNA repair gene disruptants as hosts (Ochi et al. 2011; Suzuki et al. 2017). Additionally, yeast-based genotoxicity assays incorporating DNA damage–inducible reporter constructs have been developed using three different reporter systems: multicopy (Afanassiev et al. 2000; Bui et al. 2015; Endo-Ichikawa et al. 1995; Lichtenberg-Fraté et al. 2003; Lu et al. 2015; Walmsley et al. 1997; Westerink et al. 2009) or single-copy (Jia and Xiao 2003; Ochi et al. 2011) reporter plasmids and chromosomally integrated reporter genes (Boronat and Pina 2006; Jia et al. 2002; Liu et al. 2008; Walmsley et al. 1997). Despite previous assessments of yeast-based genotoxicity assays using three different reporter systems, their responses to genotoxic agents have not been thoroughly investigated. We previously used multicopy plasmids with a 2-μm origin carrying ^P^*RNR3*-linked *lacZ*, *GFP*, and firefly luciferase genes (Ichikawa and Eki 2006; Suzuki et al. 2017), as well as a single-copy plasmid with the ^P^*RNR3*-linked *Cypridina* luciferase gene (Ochi et al. 2011). Although these yeast-based reporter assays effectively detect chemical genotoxicity, plasmid-based reporter assays present a technical challenge owing to the laborious and time-consuming handling practices associated with the auxotrophic selective medium required to maintain the reporter plasmid during culturing and genotoxicity assays. In contrast, chromosomally integrated reporter yeasts are free from these restrictions. To validate previous results obtained with different assay systems and develop practical genotoxicity assays, it is crucial to investigate the reporter gene response to genotoxic chemicals using these three different yeast assays. Furthermore, our prior observations of enhanced fold inductions (i.e., the ratio of reporter expression in the presence and absence of the chemical) following exposure to genotoxic chemicals in some DNA repair–deficient yeasts carrying a ^P^*RNR3*-linked secretory luciferase gene on a single-copy plasmid (Ochi et al. 2011) suggest the potential applications of these genotoxicity assays in studying DNA repair mechanisms in yeast.

**Fig. 1:**
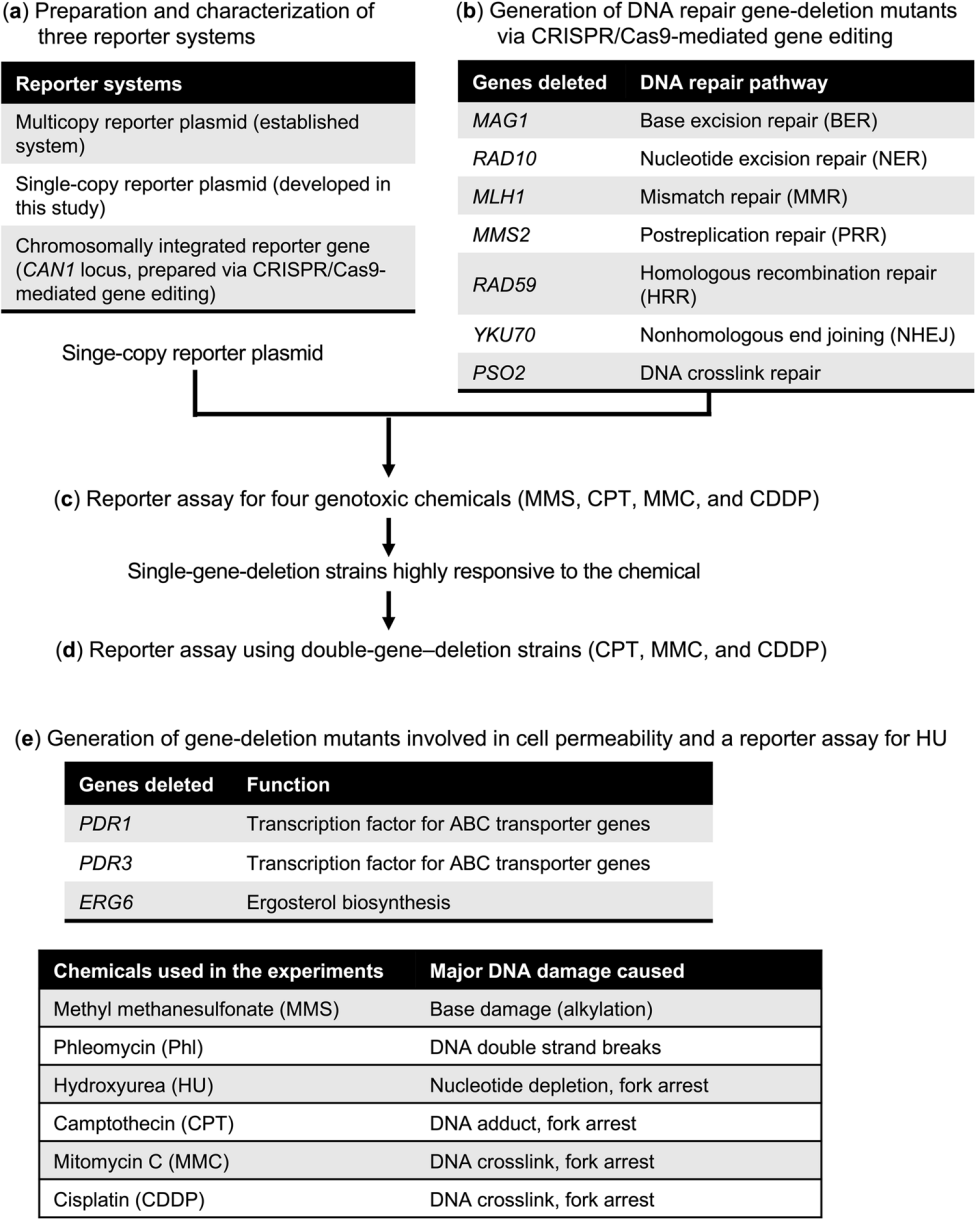
Experimental scheme of the present study. Brief descriptions and flow of five experiments (**a**–**e**) performed in this study are shown. The reporter systems tested, genes disrupted, and their functions, genotoxic chemicals, and major DNA damage caused by these chemicals are also indicated

In this study, we aimed to comparatively investigate the responses of the luciferase gene to representative genotoxic chemicals in three different yeast-based reporter systems and in single-copy reporter plasmid assays. We used systematically generated single- and double-gene-deletion mutants generated via CRISPR/Cas9-mediated gene editing (Fig. 1). Initially, yeast-based genotoxicity assays were developed using chromosomally integrated and single-copy plasmid–encoded luciferase reporter genes driven by the *RNR3* promoter, in addition to a pre-established assay using a multicopy reporter plasmid. We then investigated the response to genotoxic chemicals in three reporter systems, comparatively analyzing luciferase activity and fold induction (Fig. 1(a)). Distinct features were observed in two assays using yeast strains carrying a chromosomally integrated reporter gene and a multicopy reporter plasmid. Subsequently, we systematically generated single- or double-deletion strains of seven genes in major DNA repair pathways and three genes involved in cell permeability via CRISPR/Cas9-mediated gene disruption (Fig. 1(b, e)). For the reporter assays using DNA repair mutants, we used camptothecin (CPT), mitomycin C (MMC), and cisplatin (CDDP), whereas hydroxyurea (HU) was used for assays with cell permeability–deficient mutants. Among these anticancer chemicals, the former three were challenging to detect regarding genotoxicity using our previous reporter systems (Ichikawa and Eki 2006; Ochi et al. 2011), and the genotoxic response of HU was only evident at high concentrations, presumably due to low cell permeability in yeast (Ochi et al. 2011). We examined luciferase activity levels in these strains carrying a single-copy reporter plasmid following exposure to various genotoxic chemicals, including these anticancer drugs (Fig. 1(c–e)). Our results demonstrate that two DNA repair pathways predominantly contribute to repairing DNA crosslinks based on enhanced luciferase induction in mutants with single- and double-deletion of DNA repair genes.

## Materials and methods

### Chemicals

Methyl methanesulfonate (MMS), HU, and MMC were purchased from Sigma-Aldrich Inc. (St. Louis, MO). CPT and CDDP were obtained from FUJIFILM Wako Pure Chemical Corp. (Osaka, Japan), and phleomycin (Phl) was sourced from InvivoGen (Hong Kong). d-Luciferin potassium salt was purchased from both FUJIFILM Wako Pure Chemical Corp. and Funakoshi Co. Ltd. (Tokyo, Japan). CPT and CDDP were dissolved in dimethyl sulfoxide (FUJIFILM Wako Pure Chemical Corp.) and subsequently diluted with distilled water.

### Yeast strains

The wild-type haploid strain *S. cerevisiae* BY4741 (*MAT***a**, *his3-Δ1*, *leu2-Δ0*, *met15-Δ0*, *ura3-Δ0*) served as the parental strain for the gene disruptants prepared using CRISPR/Cas9-based gene editing. A series of 25 BY4741 strains, each carrying single- or double-gene deletions in seven DNA repair genes (*MAG1*, *MLH1*, *PSO2*, *MMS2*, *RAD10*, *RAD59*, and *YKU70*), two genes encoding transcription factors for pleiotropic drug response (*PDR1* and *PDR3*) (Mamnun et al. 2002), and one gene for ergosterol biosynthesis (*ERG6*) (Lees et al. 1995) (Table 1), were generated using CRISPR/Cas9-mediated gene editing. Six double DNA repair gene–deleted strains were also prepared from *mms2Δ*, *rad10Δ*, and *rad59Δ* strains by additional deletion of a DNA repair gene. Yeast cells were cultured at 30 °C in yeast–peptone–dextrose (YPD) media containing 1% yeast extract, 2% peptone, and 2% glucose (Dunham et al. 2015). Yeast cells carrying luciferase reporter plasmids were maintained and cultured in synthetic dextrose minimal (SD) media without histidine.

**Table 1:** Yeast strains used in this study

Strains	Genotype	Source
BY4741	*MAT***a**, *his3-Δ1*, *leu2-Δ0*, *met15-Δ0*, *ura3-Δ0*	Invitrogen
TEY001	*MAT***a**, *his3-Δ1*, *leu2-Δ0*, *met15-Δ0*, *ura3-Δ0*, *pdr1Δ*	This study
TEY002	*MAT***a**, *his3-Δ1*, *leu2-Δ0*, *met15-Δ0*, *ura3-Δ0*, *pdr3Δ*	This study
TEY003	*MAT***a**, *his3-Δ1*, *leu2-Δ0*, *met15-Δ0*, *ura3-Δ0*, *erg6Δ*	This study
TEY017	*MAT***a**, *his3-Δ1*, *leu2-Δ0*, *met15-Δ0*, *ura3-Δ0*, *mag1Δ*	This study
TEY018	*MAT***a**, *his3-Δ1*, *leu2-Δ0*, *met15-Δ0*, *ura3-Δ0*, *mlh1Δ*	This study
TEY019	*MAT***a**, *his3-Δ1*, *leu2-Δ0*, *met15-Δ0*, *ura3-Δ0*, *mms2Δ*	This study
TEY020	*MAT***a**, *his3-Δ1*, *leu2-Δ0*, *met15-Δ0*, *ura3-Δ0*, *pso2Δ*	This study
TEY021	*MAT***a**, *his3-Δ1*, *leu2-Δ0*, *met15-Δ0*, *ura3-Δ0*, *rad10Δ*	This study
TEY022	*MAT***a**, *his3-Δ1*, *leu2-Δ0*, *met15-Δ0*, *ura3-Δ0*, *rad59Δ*	This study
TEY023	*MAT***a**, *his3-Δ1*, *leu2-Δ0*, *met15-Δ0*, *ura3-Δ0*, *yku70Δ*	This study
TEY025	*MAT***a**, *his3-Δ1*, *leu2-Δ0*, *met15-Δ0*, *ura3-Δ0*, *mag1Δ*, *mms2Δ*	This study
TEY027	*MAT***a**, *his3-Δ1*, *leu2-Δ0*, *met15-Δ0*, *ura3-Δ0*, *mag1Δ*, *rad10Δ*	This study
TEY028	*MAT***a**, *his3-Δ1*, *leu2-Δ0*, *met15-Δ0*, *ura3-Δ0*, *mag1Δ*, *rad50Δ*	This study
TEY030	*MAT***a**, *his3-Δ1*, *leu2-Δ0*, *met15-Δ0*, *ura3-Δ0*, *mlh1Δ*, *mms2Δ*	This study
TEY032	*MAT***a**, *his3-Δ1*, *leu2-Δ0*, *met15-Δ0*, *ura3-Δ0*, *mlh1Δ*, *rad10Δ*	This study
TEY033	*MAT***a**, *his3-Δ1*, *leu2-Δ0*, *met15-Δ0*, *ura3-Δ0*, *mlh1Δ*, *rad59Δ*	This study
TEY035	*MAT***a**, *his3-Δ1*, *leu2-Δ0*, *met15-Δ0*, *ura3-Δ0*, *mms2Δ*, *pso2Δ*	This study
TEY036	*MAT***a**, *his3-Δ1*, *leu2-Δ0*, *met15-Δ0*, *ura3-Δ0*, *mms2Δ*, *rad10Δ*	This study
TEY037	*MAT***a**, *his3-Δ1*, *leu2-Δ0*, *met15-Δ0*, *ura3-Δ0*, *mms2Δ*, *rad59Δ*	This study
TEY038	*MAT***a**, *his3-Δ1*, *leu2-Δ0*, *met15-Δ0*, *ura3-Δ0*, *mms2Δ*, *yku70Δ*	This study
TEY039	*MAT***a**, *his3-Δ1*, *leu2-Δ0*, *met15-Δ0*, *ura3-Δ0*, *pso2Δ*, *rad10Δ*	This study
TEY040	*MAT***a**, *his3-Δ1*, *leu2-Δ0*, *met15-Δ0*, *ura3-Δ0*, *pso2Δ*, *rad59Δ*	This study
TEY042	*MAT***a**, *his3-Δ1*, *leu2-Δ0*, *met15-Δ0*, *ura3-Δ0*, *rad10Δ*, *rad59Δ*	This study
TEY043	*MAT***a**, *his3-Δ1*, *leu2-Δ0*, *met15-Δ0*, *ura3-Δ0*, *rad10Δ*, *yku70Δ*	This study
TEY044	*MAT***a**, *his3-Δ1*, *leu2-Δ0*, *met15-Δ0*, *ura3-Δ0*, *yku70Δ*, *rad59Δ*	This study
IMX672	*MAT***a**, *ura3-52*, *trp1-289*, *leu2-3,112*, *his3Δ*, *can1Δ*::*cas9-natNT2*	EUROSCRAF

### Construction of reporter plasmids

The reporter plasmids used in this study were pESC-HIS*ΔGAL1/10-*^P^*RNR3-luc2* (multicopy plasmid) and pRS313-HIS3-^P^*RNR3-luc2* (single-copy plasmid) (Table 2). The former was prepared in a previous study (Suzuki et al. 2017), whereas the latter was constructed using pRS313 DNA amplified from pGEV-HIS3 DNA (Gao and Pinkham 2000) through inverse polymerase chain reaction (PCR) with KOD FX Neo DNA polymerase (Toyobo, Tokyo) and the primers pRS313-*Bam*-invF and pRS313-*Not*-invR (Table S1). The reporter gene cassette of the *ADH1* terminator (^T^*ADH1*)-^P^*RNR3*-luciferase (*luc2*) gene-*CYC1* terminator (^T^*CYC1*) was PCR-amplified using pESC-HIS*ΔGAL1/10-*^P^*RNR3-luc2* DNA as a template with primers pRS313-*Not*-TRX2yNlucP-IFF and pRS313-*Bam*-TRX2yNlucP-IFR. The resulting PCR product was purified using a NucleoSpin Gel and PCR Clean-up Kit (Takara, Osaka) and cloned into pRS313 DNA using the In-Fusion Snap Assembly Kit (Takara). The resulting plasmid DNA, named pRS313-HIS3-^P^*RNR3-luc2*, was purified using the NucleoSpin Plasmid Easy Pure Kit (Takara) and sequenced by Macrogen Japan (Tokyo, Japan). Sequence data were assembled and analyzed using ATGC and Genetyx software (version 13; Genetyx Co., Tokyo), respectively. Oligo-DNAs were synthesized by FASMAC (Atsugi, Japan) and Integrated DNA Technologies (Coralville, IO).

**Table 2:** Plasmids used in this study

Plasmid	Description
pRS415	*ARS4/CEN6* plasmid vector with *LEU2* marker (Stratagene)
pRS415-LEU-Cas9	pRS415 plasmid containing *cas9* expression cassette DNA at the *Sma* I site (this study)
pMEL10	RNA expression plasmid with *URA3* marker and guide RNA (gRNA) for *CAN1* gene (Mans et al. 2015) and obtained from EUROSCARF
pMEL10-*mag1*	pMEL10 derivative for gRNA expression of* MAG1*
pMEL10-*mlh1*	pMEL10 derivative for gRNA expression for *MLH1* gene
pMEL10*-mms2*	pMEL10 derivative of gRNA expression of *MMS2*
pMEL10-*pso2*	pMEL10 derivative of gRNA expression of *PSO2*
pMEL10-*rad10*	pMEL10 derivative of gRNA expression of *RAD10*
pMEL10-*rad59*	pMEL10 derivative of gRNA expression of *RAD59*
pMEL10-*yku70*	pMEL10 derivative of gRNA expression of *YKU70*
pMEL10-*pdr1*	pMEL10 derivative of gRNA expression of *PDR1*
pMEL10-*pdr3*	pMEL10 derivative of gRNA expression of *PDR3*
pMEL10*-erg6*	pMEL10 derivative of gRNA expression of *ERG6*
pESC-HIS*ΔGAL1/10-*^P^*RNR3-luc2*	Multicopy luciferase reporter plasmid driven by *RNR3* promoter (Suzuki et al. 2017)
pGEV-HIS3	Template plasmid used of preparing pRS313 DNA, kindly provided by Dr. Pinkham (Gao and Pinkham 2000)
pRS313-HIS3-^P^*RNR3-luc2*	Single-copy luciferase reporter plasmid driven by *RNR3* promoter

### CRISPR/Cas9-mediated gene disruptions

The yeast strains with single- or double-gene deletions were derived from the parent strain BY4741 through CRISPR/Cas9-based gene editing following a previously described method (Mans et al. 2015) with modifications. Briefly, yeast cells carrying the Cas9-expression plasmid pRS415-LEU-Cas9 were cotransformed with the pMEL10-derived gRNA expression plasmid and a target gene-specific repair DNA fragment. The resulting *LEU*^+^- and *HIS*^+^-transformants were assessed using colony PCR with allele-specific primer sets (Table S1) and KOD FX Neo polymerase to confirm allele deletion. The target gene, i.e., an open reading frame (ORF), was replaced by a repair DNA fragment connected with target gene’s 3ʹ- and 5ʹ-flanking DNAs, resulting in the corresponding gene-deletion strain. The gene-deletion strains, in which the target locus was repaired by the repair DNA fragment through homologous recombination, could only survive on selective SD agar plates without leucine and histidine.

The plasmid pRS415-LEU-Cas9 was prepared as follows. First, the *Cas9* gene was PCR-amplified using PrimeSTAR HS polymerase (Takara) and genomic DNA prepared from the yeast strain IMX672 (Mans et al. 2015) with primers p414-2873dIFF and p414-4653dIFR. Subsequently, it was cloned into the *Sma* I site of pRS415 DNA using the In-Fusion HD Cloning Kit (Takara). The gRNA expression plasmids for the target gene were then generated from pMEL10 containing the *CAN1*-targeted gRNA sequence via inverse PCR with primers #6005_p426CRISPR rv2 and target gene_#6006_p426CR fw2 (Table S1) using PrimeSTAR MAX DNA polymerase (Takara). The resulting pMEL10 derivatives, in which the *CAN1* gRNA sequence was replaced with the target gene gRNA sequence, were used for target gene-deletion through cotransformation with the target gene’s repair DNA fragment. Repair fragments, consisting of a 130–240-bp length of the target gene’s 5ʹ- and 3ʹ-flanking DNAs, were PCR-amplified from BY4741 genomic DNA with KOD FX Neo polymerase and the corresponding primer sets (Table S2). These fragments were then connected via PCR with forward and reverse primers for the 5ʹ- and 3ʹ-flanking DNAs. To connect both flanking DNAs, reverse primers for the 5ʹ-flanking DNA were attached via the tail sequence of the 3ʹ-flanking sequence. The resulting repair DNA fragment (350–470 bp) was used for gene disruption. Deletion of the target gene in the disruptant was confirmed via colony PCR spanning the ORF, with PCR products from the wild-type and deletion strains clearly distinguishable by size (Table S3). We disrupted seven genes in a major DNA repair pathway (*MAG1*, *MLH1*, *MMS2*, *PSO2*, *RAD10*, *RAD59*, and *YKU70*), two genes encoding transcription factors regulating ABC transporter gene expression (*PDR1* and *PDR3*), and *ERG6*, a gene involved in ergosterol biosynthesis. Additionally, double-gene-deletion strains with *mms2Δ*, *rad10Δ*, and *rad59Δ* alleles were prepared by deleting an additional DNA repair gene. The double-gene-deletion strains with the *rad59Δ* allele were generated from each single-gene-deletion strain by additionally disrupting *RAD59*, given that Rad59p plays a role in homologous recombination (Symington 2002), which is crucial for CRISPR/Cas9-based gene disruption. The deleted allele was confirmed via colony PCR using the primer set for a target gene (Table S3). The mutated cells were cultured in nonselective YPD medium for 1–3 days to remove plasmid DNA and then plated on YPD agar plates to isolate colonies. These clones were confirmed to lack the auxotrophic markers of the plasmid through their inability to grow on the SD selection agar plates (ForMedium, Hunstanton, UK).

### Development of a yeast strain with a chromosomally integrated luciferase reporter gene

A yeast strain with a chromosomally integrated luciferase reporter gene was generated from the wild-type BY4741 strain by integrating the ^T^*ADH1*-^P^*RNR3*-*luc2*-^T^*CYC1* cassette, connected with the 5ʹ- and 3ʹ-flanking DNA of the *CAN1* gene at each end, into the *CAN1* locus using CRISPR/Cas9. Briefly, the ^T^*ADH1*-^P^*RNR3*-*luc2*-^T^*CYC1* cassette (approximately 3.3 kb) was amplified from pESC-HIS*ΔGAL1/10-*^P^*RNR3-luc2* using KOD FX Neo polymerase and primers TADH1-5F2-25CAN1-Rtail and TCYC1-3R3-25CAN1-Ftail. The 5ʹ- and 3ʹ-DNA flanking *CAN1* (190 and 241 bp, respectively) were amplified using KOD FX Neo polymerase and the primer sets 5ʹ-CAN1-F and 5-CAN1-R_5F2_30tail and 3-CAN1-F_3R3_30tail and 3ʹ-CAN1-R, respectively. Each flanking DNA was then fused to the corresponding end of the ^T^*ADH1*-^P^*RNR3*-*luc2*-^T^*CYC1* cassette DNA via PCR using PrimeSTAR DNA polymerase (Takara) and primers 5ʹ-CAN1-F and 3ʹ-CAN1-R. Yeast cells with pRS415-LEU-Cas9 were cultured in SD medium without leucine and cotransformed with pMEL10 expressing *CAN1*-targeted gRNA and the ^T^*ADH1*-^P^*RNR3*-*luc2*-^T^*CYC1* cassette DNA fused with both flanking DNAs. The resulting *LEU*^+^- and *HIS*^+^-clones were tested for the absence of the *CAN1* ORF and the presence of the reporter cassette DNA at the *CAN1* locus using colony PCR, with primers CAN1-5check-F and CAN1orf-R used for *CAN1*’s 5ʹ-region, CAN1orf-F and CAN1_dg rv used for *CAN1*’s 3ʹ-region for *CAN1* gene, and CAN1-5check-F and luc2-SQR2 used for the integrated reporter gene (Table S1).

### Yeast transformation

Yeast cells underwent transformation with reporter plasmids using a lithium acetate protocol (Gietz et al. 1992). Transformants were selected on SD medium without histidine (ForMedium), and independent colonies were streaked onto fresh selection agar plates prior to their use. For gene disruption, yeast cells were initially transformed using the Cas9-expression plasmid pRS415-LEU-Cas9 with the *LEU2* marker. Subsequently, the *LEU*^+^-transformants were cotransformed using the pMEL10-derived gRNA expression plasmid with the *HIS3* marker and a repair DNA fragment corresponding to a target gene. The gene-deletion strains were selected on SD agar plates lacking leucine or histidine.

### Luciferase assay

Luciferase activity was assessed in yeast containing luciferase reporter plasmids exposed to tested agents, following a previously established protocol (Suzuki et al. 2017) with several modifications. Briefly, yeast cells were grown on SD agar plates lacking histidine and cultured with 10 mL of histidine-free SD medium in a 50-mL conical tube at 30 °C with continuous shaking for 20–40 h. Yeast cells containing the chromosomally integrated luciferase reporter were cultured with 10 mL of YPD medium. The yeast cells were collected using centrifugation and resuspended in YPD medium to achieve an approximate OD_600_ of 1.0. Subsequently, 100 µL of yeast suspension was inoculated per well in triplicate in a 96-well white plate (Coster, No. 3912), and the agents to be tested were added at the specified concentrations. The yeast cells in the microplate were then incubated at 30 °C for 8 h under saturated humidity. Following the addition of 100 µL of YPD medium, 100 µL aliquots of the diluted cell suspension were transferred to a new 96-well white plate to measure the absorbance at 600 nm (*A*_600_) and luciferase activity using a multimode plate reader (Tecan Infinite M1000). d-Luciferin (0.5 mM) was introduced, and the luminescence and *A*_600_ value of the yeast culture in the plate were measured after 30 min. The luciferase activity is expressed in arbitrary units, defined as the luminescence (counts) in 1 s per *A*_600_. The fold induction was calculated as the ratio of luciferase activity in the presence and absence of each test chemical. Data were analyzed in Microsoft Excel using a two-tailed paired Student *t*-test via the T.TEST function, and *p* < 0.01 was considered statistically significant.

## Results

### Development of a yeast-reporter strain with a *RNR3* promoter–driven chromosomally integrated luciferase gene, and comparative characterization of the reporter response to genotoxic chemicals using plasmid–carrying strains

We developed yeast-reporter strains containing pESC-HIS-derived multicopy plasmids with the *RNR3* promoter (^P^*RNR3*)–linked *GFP* and modified firefly luciferase (*luc2*) genes. Subsequently, yeast-based assays effectively detected chemical genotoxicity. However, these yeast-reporter strains required the auxotrophic selective medium lacking histidine for culturing and assaying, given the need to maintain the reporter plasmid with the *HIS3* marker. Consequently, we employed CRISPR/Cas9-based gene editing to replace *CAN1* with the ^P^*RNR3*-driven *luc2* gene, resulting in a yeast-reporter strain easily maintained with nonselective YPD medium.

**Fig. 2:**
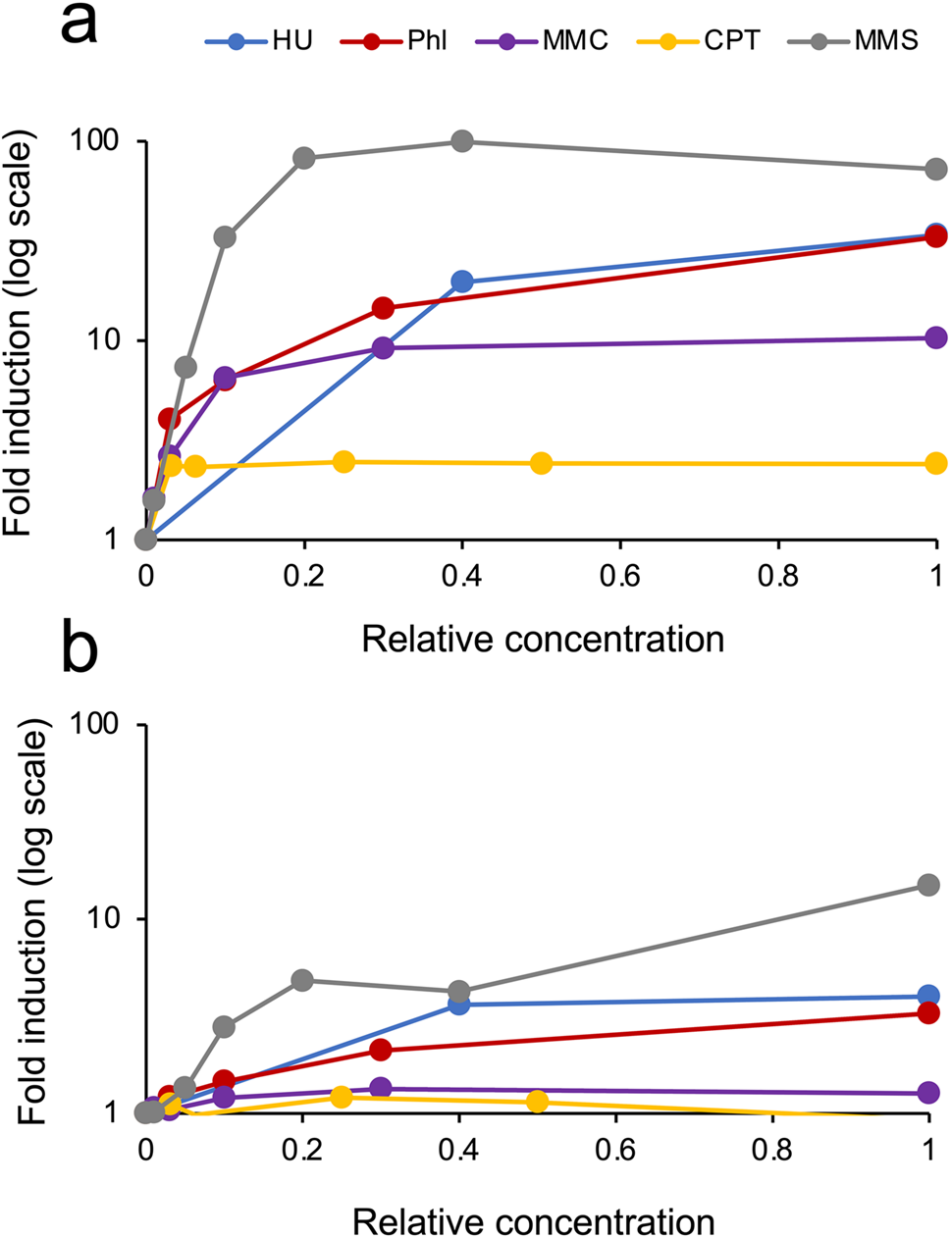
Fold induction of luciferase activity induced by five genotoxic chemicals in yeast cells with a chromosomally integrated and a multicopy plasmid–based reporter gene. Log scale fold induction values for the yeast BY4741 strain with an integrated *RNR3* promoter–linked luciferase reporter gene at the *CAN1* locus (**a**) and with the multicopy reporter plasmid (**b**) after an 8-h exposure to the indicated relative concentrations (relative to the highest concentration used as 1.0) of hydroxyurea (HU), phleomycin (Phl), mitomycin C (MMC), camptothecin (CPT), and methyl methanesulfonate (MMS), the highest concentrations of which were 50 mM, 10 μg/mL, 300 μM, 160 μg/mL, and 0.05% (w/v), respectively

We investigated luciferase activity levels in the yeast cells with a multicopy reporter plasmid and those with a chromosomally integrated reporter following exposure to five genotoxic chemicals: HU, MMS, Phl, MMC, and CPT. Dose–response bar charts, excluding CPT, showed increased luciferase activities in both types of reporter yeast cells. However, luminescence intensity significantly differed between the two reporter strains. The pre-established assay, employing a multicopy plasmid, exhibited approximately 10–100-fold higher intensities compared with the chromosomally integrated reporter strain (Fig. S1a–e). Nevertheless, the luciferase fold induction was markedly higher in the chromosomally integrated reporter strains than in the pre-established plasmid reporter strain (Fig. S1f–j); for instance, the former strain treated with 0.02% MMS showed a 25-fold higher fold induction (Fig. S1g). Fold inductions (log scale) for each chemical in the assays using a chromosomally integrated reporter and a multicopy reporter plasmid are summarized with relative concentrations (relative to the highest concentration tested as 1.0) in Fig. 2a and b, respectively. The pre-established assay detected genotoxicity induced by MMS (15-fold), HU (fourfold), and Phl (threefold) with the highest fold induction but exhibited weak detection of MMC- and CPT-induced genotoxicity (Fig. 2b). In contrast, the chromosomally integrated reporter yeast assay displayed high fold inductions (> 10) with all chemical treatments except for CPT (twofold induction; Fig. 2a).

**Fig. 3:**
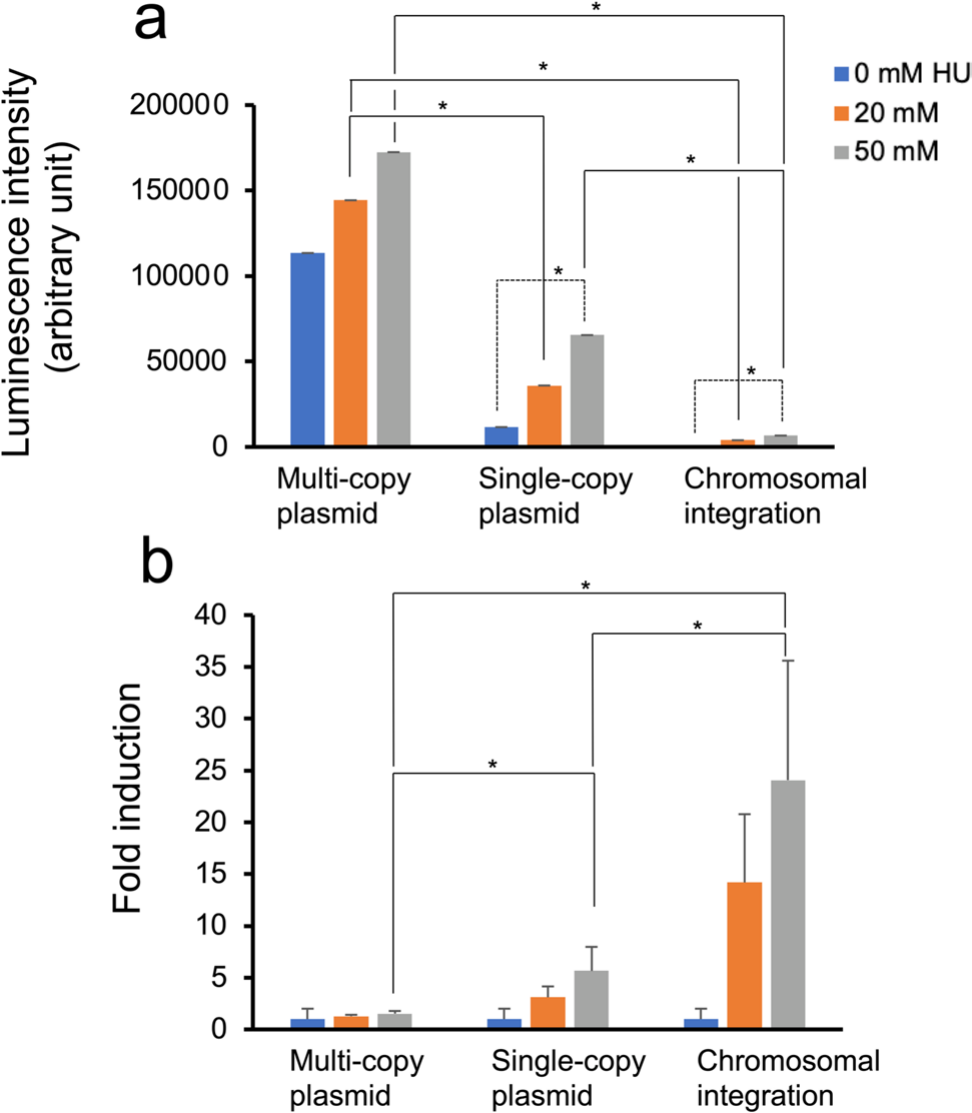
Expression levels in the BY4741 yeast strain carrying a multi- and a single-copy reporter plasmid and a chromosomally integrated reporter gene driven by the *RNR3* promoter following hydroxyurea exposure. The BY4741 strain with a multicopy plasmid pESC-HIS*ΔGAL1/10*-^P^*RNR3-luc2*, a single-copy plasmid pRS313-HIS3-^P^*RNR3-luc2*, and a chromosomally integrated.^P^*RNR3-luc2* gene at the *CAN1* locus was cultured with (20 or 50 mM) or without hydroxyurea for 8 h. Chemiluminescence intensity derived from luciferase per *A*_600_-normalized cells (**a**) and fold induction (**b**) are shown with standard deviations. Significant pairs based on Student’s *t*-test (*p* < 0.01) are indicated by asterisks (Table S4)

We also developed a yeast strain containing a pRS313-derived single-copy luciferase reporter plasmid owing to its ease of preparation compared with chromosomally integrated reporter yeasts. We examined luciferase activity levels in three reporter strains with a multicopy or single-copy reporter plasmid or a chromosomally integrated reporter gene after treatment with 20 and 50 mM HU for 8 h as a representative genotoxicant. Observations of the multicopy plasmid and chromosomally integrated reporter gene–containing strains (Fig. 3) were consistent with those shown in Fig. 2, with high and low activity levels (Fig. 3a) and high and low fold inductions (Fig. 3b) in the former and latter strains, respectively. The luciferase levels significantly differed between strains harboring a multicopy reporter plasmid and those with a chromosomally integrated reporter gene in the presence of HU (*t*-test, *p* < 0.01; Table S4). Significant induction of luciferase activity was observed between yeasts with a multicopy reporter plasmid and those with a single-copy reporter plasmid following a 20-mM HU treatment. Similarly, a significant induction was noted between yeast strains with a single-copy reporter plasmid and those with a chromosomally integrated reporter gene following a 50-mM HU treatment (Fig. 3a). Fold inductions in the chromosomally integrated reporter strain were significantly higher than those in the other two reporter strains treated with 50 mM HU (Fig. 3b), with standard deviations in fold induction also tending to be larger in the chromosomally integrated reporter strain. The strain with a single-copy reporter plasmid exhibited an intermediate reporter response, with luciferase levels lower than those in the assay with a multicopy reporter plasmid but markedly higher than those in the assay with a chromosomally integrated reporter strain (Fig. 3a), whereas fold inductions showed the opposite pattern (Fig. 3b).

### Response of the luciferase gene in single DNA repair gene–deleted strains treated with four genotoxic chemicals

**Fig. 4:**
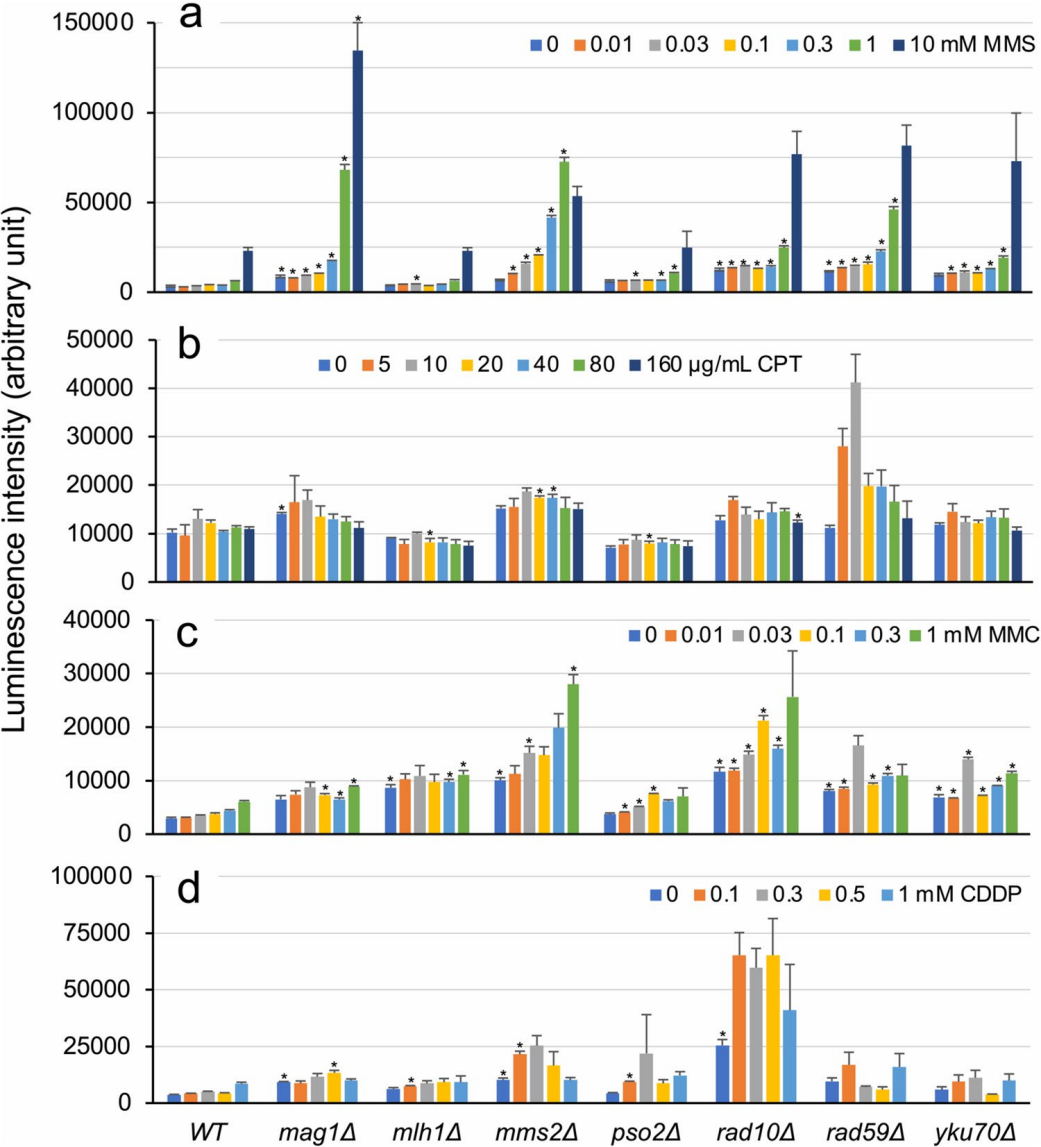
Luciferase activity levels induced by four genotoxic chemicals in the wild-type and seven DNA repair gene–deleted BY4741 strains carrying a single-copy luciferase reporter plasmid. The wild-type and seven single-gene-deletion strains carrying pRS313-HIS3-^P^*RNR3-luc2* were cultured in YPD medium with or without the indicated concentrations of MMS (**a**), CPT (**b**), MMC (**c**), and CDDP (**d**) for 8 h. Luciferase-mediated chemiluminescence intensity is shown with standard deviations. Bar plots with asterisks indicate mutant cells with significant luminescent intensity compared with the corresponding wild-type cells (Student’s *t*-test, *p* < 0.01; Table S5)

**Fig. 5:**
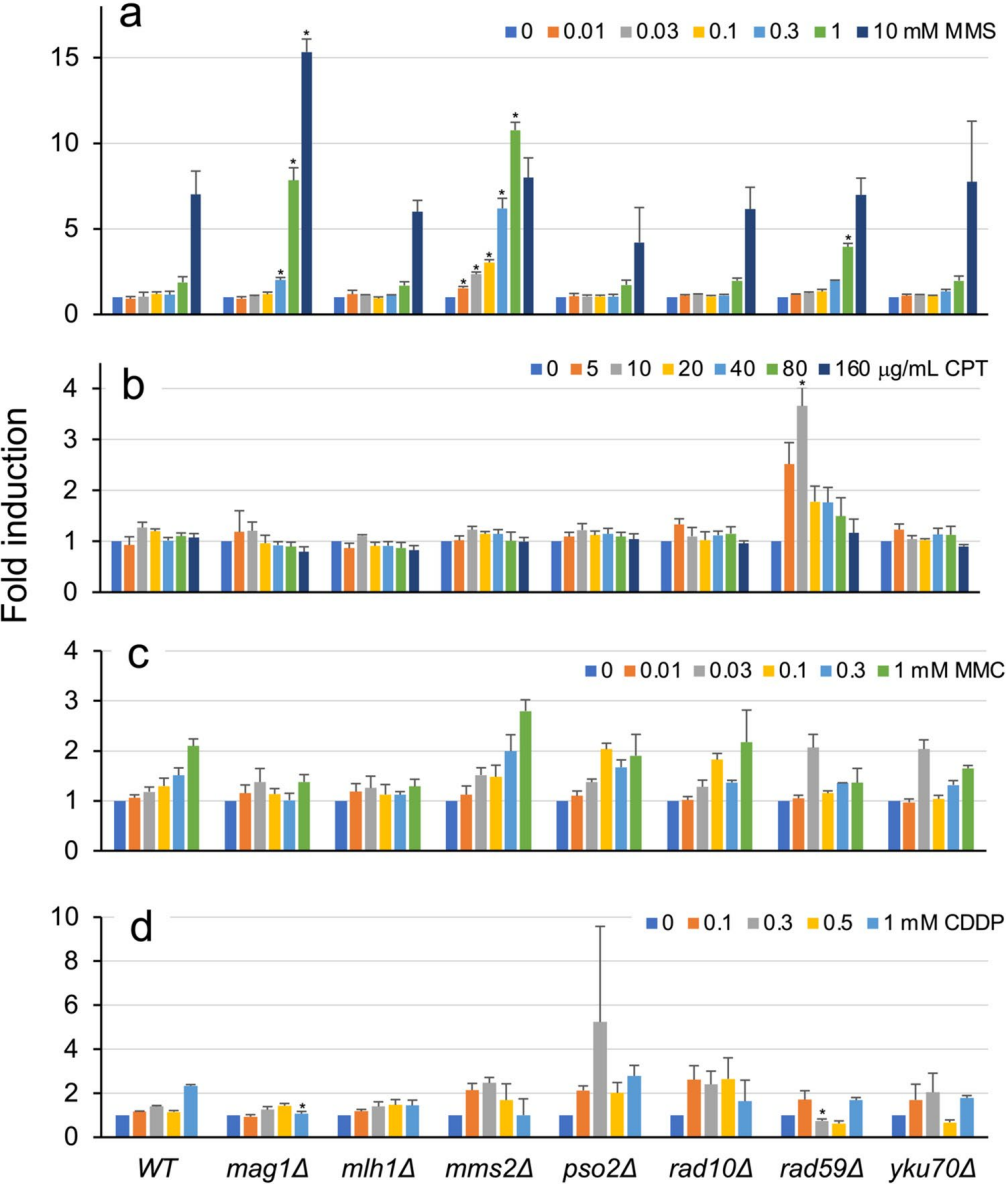
Fold induction of luciferase activity in the wild-type and seven DNA repair single-gene-deletion strains treated with four genotoxic chemicals. The wild-type and seven single-gene-deletion BY4741 strains carrying pRS313-HIS3-^P^*RNR3-luc2* were cultured in YPD medium with or without the indicated concentrations of MMS (**a**), CPT (**b**), MMC (**c**), and CDDP (**d**) for 8 h. Fold induction of luciferase activity is shown with standard deviations. Bar plots with asterisks indicate mutant cells with significant luminescent intensity compared with the corresponding wild-type cells (Student’s *t*-test, *p* < 0.01; Table S5)

We used CRISPR/Cas9-mediated gene editing to systematically generate deletion strains of seven DNA repair genes in major DNA repair pathways: *MAG1* in base excision repair (BER), *MLH1* in mismatch repair (MMR), *MMS2* in postreplication repair (PRR), *PSO2* in crosslink repair, *RAD10* in nucleotide excision repair, *RAD59* in homologous recombination repair (HRR), and *YKU70* in nonhomologous end joining (NHEJ) (Fig. 1). Subsequently, we investigated the luciferase gene’s response after an 8-h exposure to the genotoxic agents MMS, CPT, MMC, and CDDP, including three anticancer drugs. The activities induced in the wild-type and seven gene-deletion strains carrying a single-copy reporter plasmid in the presence of various concentrations of these chemicals are shown in Fig. 4. In MMS assays, luciferase activities in the *mag1Δ*, *mms2Δ*, *rad10Δ*, *rad59Δ*, and *yku70Δ* strains were higher than those in wild-type cells (Fig. 4a). Enhanced luciferase induction was observed in the *rad59Δ* strain treated with 5 and 10 μg/mL CPT (Fig. 4b), as well as in the *mms2Δ* and *rad10Δ* strains treated with MMC (Fig. 4c) and CDDP (Fig. 4d), respectively. However, significantly higher fold inductions were only detected in MMS-treated *mag1Δ*, *mms2Δ*, and *rad59Δ* strains (Fig. 5a), as well as in the CPT-treated *rad59Δ* strain (Fig. 5b), compared with the corresponding wild-type strains (*t*-test, *p* < 0.01; Table S5). Interestingly, the background luciferase activity levels in the absence of chemicals increased in several deletion strains in four independent experiments (Fig. S2; Table S6). Despite some differences among the experiments, enhanced luciferase activities were predominantly found in several DNA repair–deficient mutants, such as strains *mag1Δ* and *rad10Δ* (three out of four experiments), in addition to *mms2Δ* and *rad59Δ* (two out of four), leading to inconsistent results between the luciferase activity and fold induction in assays employing these mutants. Nevertheless, although high luciferase activity levels were detected, the resultant fold induction was not significantly increased, given that fold induction is considered the activity ratio in chemical-treated cells relative to untreated cells.

### Response of the luciferase gene in the reporter strains with double-deleted DNA repair genes after treatment with CPT or DNA crosslinkers

We examined four deletion strains that exhibited higher luciferase activities following exposure to CPT (*rad59Δ*), MMC (*mms2Δ*), and CDDP (*mms2Δ* and *rad10Δ*), given the genotoxicity of these anticancer drugs. This emphasis is particularly pertinent as CDDP is difficult to detect using our pre-established reporter assays with wild-type yeast cells (Ochi et al. 2011). Therefore, we generated a series of double-deletion strains from these strains to investigate luciferase activities following chemical treatments.

**Fig. 6:**
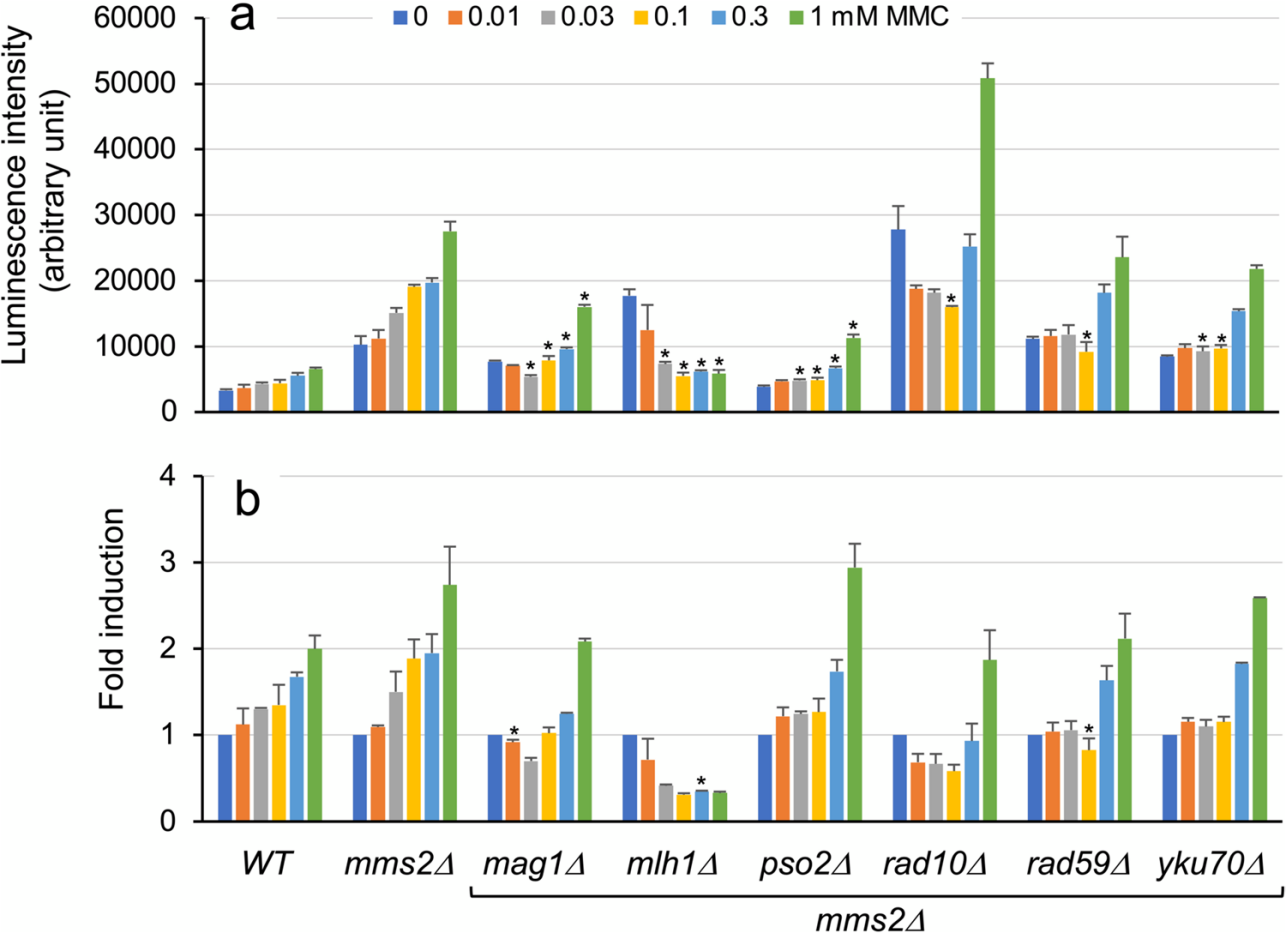
Luciferase induction with MMC in yeast DNA repair double-gene-deletion mutants with the *mms2Δ* allele. Wild-type BY4741, *mms2Δ*, and six *mms2Δ*-derived double-gene-deletion mutants with plasmid pRS313-HIS3-^P^*RNR3-luc2* were cultured with or without the indicated concentrations of MMC for 8 h. Luciferase activity (**a**) and fold induction (**b**) in cells are shown with standard deviations. Bar plots with asterisks indicate double-gene-deletion mutant cells with significant luminescent intensity compared with the corresponding *mms2Δ* cells (Student’s *t*-test, *p* < 0.01; Table S8)

**Fig. 7:**
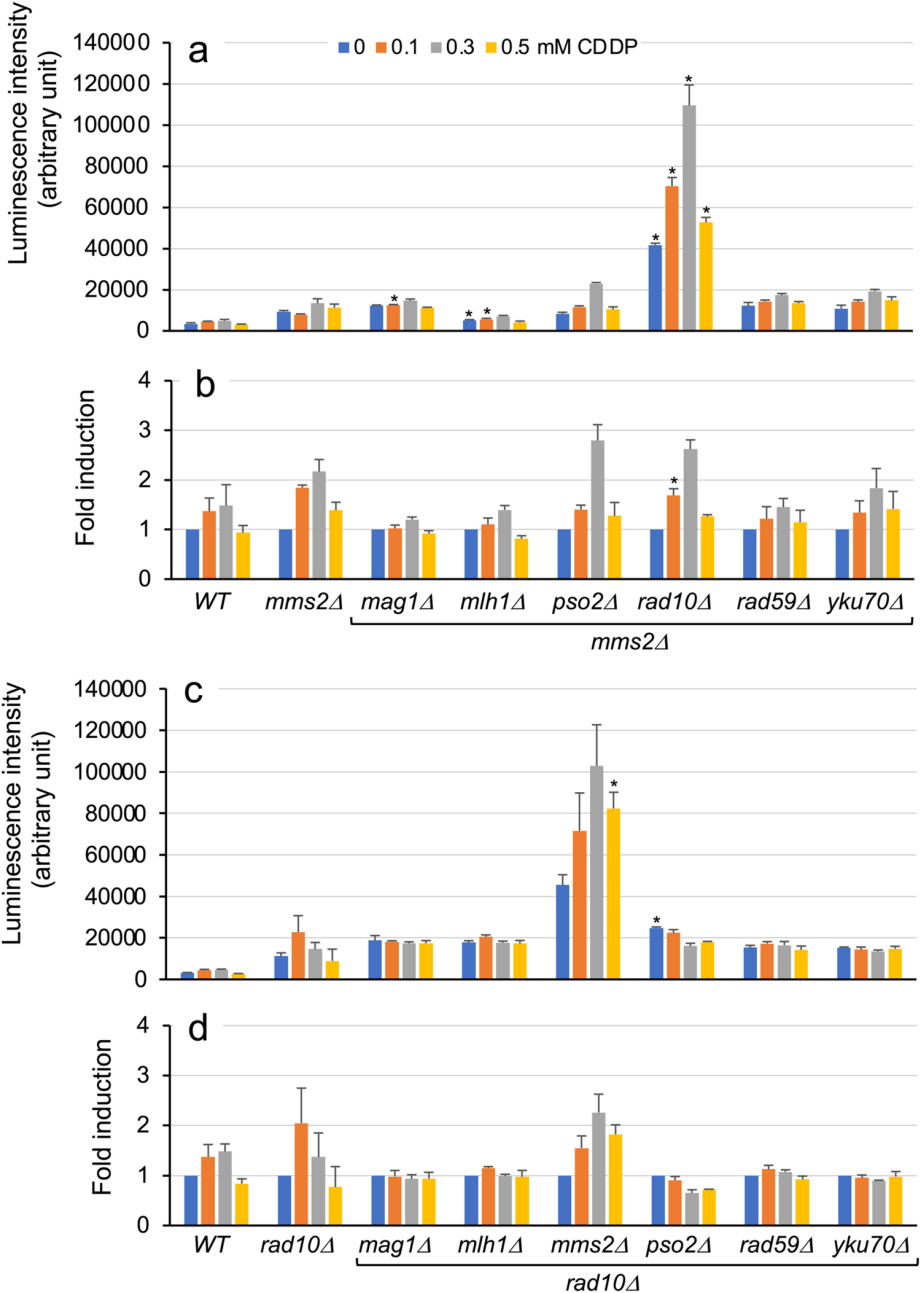
Luciferase induction with CDDP in yeast DNA repair double-gene-deletion mutants with the *mms2Δ* and *rad10Δ* alleles. Wild-type BY4741, *mms2Δ*, and six *mms2Δ*-derived double-gene-deletion mutants (**a**, **b**) along with *rad10Δ* and six *rad10Δ*-derived double-gene-deletion mutants (**c**, **d**) with plasmid pRS313-HIS3-^P^*RNR3-luc2* were cultured with or without the indicated concentrations of CDDP for 8 h. Luciferase activity (**a**,** c**) and fold induction (**b**, **d**) in cells are shown with standard deviations. Bar plots with asterisks indicate double-gene-deletion mutant cells with significant luminescent intensity compared with the corresponding parent strains (Student’s *t*-test, *p* < 0.01; Table S9)

Initially, we measured the luciferase activities in the wild-type and *rad59Δ* strains as well as six double-deletion strains with the *rad59Δ* allele carrying a single-copy reporter plasmid following treatment with low CPT concentrations (Fig. S3). However, none of the six double-deletion strains exhibited luciferase activity levels higher than those of their parent strain *rad59Δ* (Table S7). Subsequently, we assessed luciferase activity following exposure to five different MMC concentrations using six *mms2Δ*-derived double-deletion strains together with the wild-type and their parent strains. Although none of the double-deletion strains exhibited enhanced fold induction compared with their parent strain, a higher luciferase activity level was detected in the *rad10Δ*/*mms2Δ* strain treated with 1 mM MMC compared with the corresponding *mms2Δ* cells (Fig. 6; Table S8). Finally, luciferase activities of the wild-type, *mms2Δ*, and *rad10Δ* strains, as well as their 11 double-deletion strains with a reporter plasmid, were tested following exposure to 0.1, 0.3, and 0.5 mM CDDP (Fig. 7; Table S9). Significantly higher luciferase activity levels were detected in the *rad10Δ*/*mms2Δ* reporter strain compared with the parent *mms2Δ* strain (Fig. 7a), although fold induction values remained comparable between these strains owing to the enhanced basal luciferase expression level without CDDP treatment (Fig. 7b). Results were consistent in assays using *rad10Δ*-derived double-deletion strains, where the *mms2Δ*/*rad10Δ* strain exhibited markedly high luciferase activity levels, in contrast to the other five double-deletion strains (Fig. 7c).

### Response of the luciferase gene to HU treatment in strains with deletions of cell permeability–related genes

We prepared three gene-deletion strains (*pdr1Δ*, *pdr3Δ*, and *erg6Δ*) with defects in cell permeability and investigated HU-induced luciferase activities in these strains carrying a single-copy reporter plasmid. Pdr1p and Pdr3p form a transcription factor that regulates the expression of ABC transporter (drug efflux pump) genes (Mamnun et al. 2002), and Erg6p is a methyltransferase involved in ergosterol biosynthesis, altering the ergosterol composition of the plasma membrane (Lees et al. 1995). HU was used as a DNA-damaging chemical to evaluate cell permeability in mutants because effective HU concentrations are 10–100-fold higher than those in cultured human cells. For example, the HU concentrations required for cell synchronization in human and budding yeast cells are 2 mM (Biegel et al. 1987) and 200 mM (Rosebrock 2016), respectively. Although the induction levels in the two *PDR*-deletion strains were comparable to those in the parental strains, enhanced luciferase activity and fold induction were observed in the *erg6Δ* strain (Fig. S4; Table S10).

## Discussion

Yeast-based bioassays using reporter genes linked to DNA damage–inducible promoters are valuable for assessing mutagenicity due to genotoxic chemicals (Eki 2018). We previously used yeast cells with ^P^*RNR3*-driven reporter plasmids containing a 2-μm origin and the *HIS3* auxotrophic marker to detect genotoxicity (Suzuki et al. 2017). Various studies have employed multicopy reporter plasmids with assays driven by promoters such as ^P^*RAD54* (Afanassiev et al. 2000; Billinton et al. 1998; Bui et al. 2016; Cahill et al. 2004; Knight et al. 2007, 2002; Lichtenberg-Fraté et al. 2003; Van Gompel et al. 2005; Walmsley et al. 1997; Walsh et al. 2005; Westerink et al. 2009), ^P^*RNR2* (Afanassiev et al. 2000; Lu et al. 2015; Walsh et al. 2005), ^P^*RNR3* (Endo-Ichikawa et al. 1995), and ^P^*PLM2* and ^P^*DIN7* (Bui et al. 2015). A chromosomally integrated reporter system has also been used with *RNR3* (Boronat and Pina 2006; Jia et al. 2002; Wei et al. 2013), *RAD51* (Liu et al. 2008), *HUG1* (Benton et al. 2007, 2008; Wei et al. 2013), and *RAD54* (Boronat and Pina 2006; Walmsley et al. 1997) promoter-linked reporter assays for genotoxicity detection. Two ^P^*RNR3*-linked single-copy *lacZ* and *Cypridina* luciferase gene reporter plasmids, namely pZZ2 (Zhou and Elledge 1992) and pCLY-*RNR3* (Ochi et al. 2011), were reported to enhance genotoxicity testing with yeast mutants (Jia and Xiao 2003; Ochi et al. 2011; Zhang et al. 2010, 2008, 2011). Given that three different yeast-based reporter systems were employed in previous studies, it has become crucial to determine reporter responses to DNA-damaging agents in these systems to assess potential differences in their results. Therefore, in the present study, we developed yeast strains containing an *ARS/CEN*-plasmid-based and chromosomally integrated ^P^*RNR3*-driven luciferase gene (*luc2*), subsequently comparing reporter gene responses upon exposure to genotoxic chemicals in three reporter systems, including a multicopy plasmid–based reporter assay. Chromosomally integrated reporter yeasts exhibited a contrasting induction of luciferase activity due to HU-induced DNA damage compared with the pre-established multicopy plasmid–based reporter yeasts (Fig. 3), with high fold induction and low luciferase levels in the former and low fold induction and high luciferase levels in the latter. Despite displaying low luciferase activity, the significant increase in the luciferase activity ratio with induction observed in a chromosomally integrated reporter assay compared with the pre-established assay system indicates a robust genotoxic-dependent induction ratio in this system. Regarding sensitivity to genotoxicity, the lowest effective MMS concentration (0.0025%) detected in the yeast-reporter assay using a chromosomally integrated gene was comparable to the concentrations in plasmid-based reporter assays conducted in our previous studies (Ichikawa and Eki 2006; Ochi et al. 2011; Suzuki et al. 2017) (Fig. S2). However, this system may be improved for sensitive genotoxicity detection using a genetically designed luciferase expressing stronger chemiluminescence, such as NanoLuc luciferase (Masser et al. 2016; Shichinohe et al. 2023). Contrasting fold inductions (i.e., the signal-to-background activity ratio) between the chromosomally integrated reporter and reporter plasmid-based assay systems may be attributed to different gene copy numbers and/or molecular environments, such as chromatin structure, which differ with reporter gene transcriptional activation in the plasmid DNA or chromosomal DNA transcriptional activation. Understanding the reason behind the observed high fold inductions in chromosomally integrated reporter assays remains challenging. Yeast strains with a single-copy reporter plasmid exhibited an intermediate response compared with the two types of reporter yeast cells. Despite both yeast strains carrying an equal copy number of the reporter gene, the luciferase levels induced by HU in the yeast with a reporter plasmid were substantially higher than those in the chromosomally integrated reporter yeast (Fig. 3). This may be explained by different molecular environments around the reporter gene as described above.

The response of the luciferase gene to the alkylating agent MMS was readily detected in both chromosomally integrated and multicopy plasmid–based reporter yeasts in terms of fold induction (Fig. 2). However, the luciferase reporter gene exhibited poor responsiveness to two antitumor agents, the DNA crosslinker MMC (Tomasz 1995) and topoisomerase I inhibitor CPT (Khaiwa et al. 2021), in yeasts carrying a multicopy reporter plasmid. Despite this, the DNA damage induced by these chemicals was successfully detected in the chromosomally integrated reporter yeasts (Fig. 2a). Thus, we have clarified the characteristics of three reporter systems: a yeast-based reporter assay using a chromosomally integrated luciferase gene can detect the genotoxicity of chemicals that are challenging to detect using established plasmid-based reporter assays. Moreover, yeast cells with a chromosomally integrated reporter gene can be maintained in nonselective nutrient medium, such as YPD, facilitating the easy and convenient handling of yeast cells in a genotoxicity assay. However, this reporter system faces challenges related to low levels of reporter expression and more time-consuming and laborious preparation of chromosomally integrated reporter yeasts compared with plasmid-based reporter yeasts. Conversely, plasmid-based assays exhibit high levels of reporter expression and are well-suited for generating a large number of yeast mutants carrying a reporter plasmid through transformation in a short period. Consequently, the selection of a reporter assay can be tailored to the intended purpose based on the distinctive features of reporter response in each system.

**Fig. 8:**
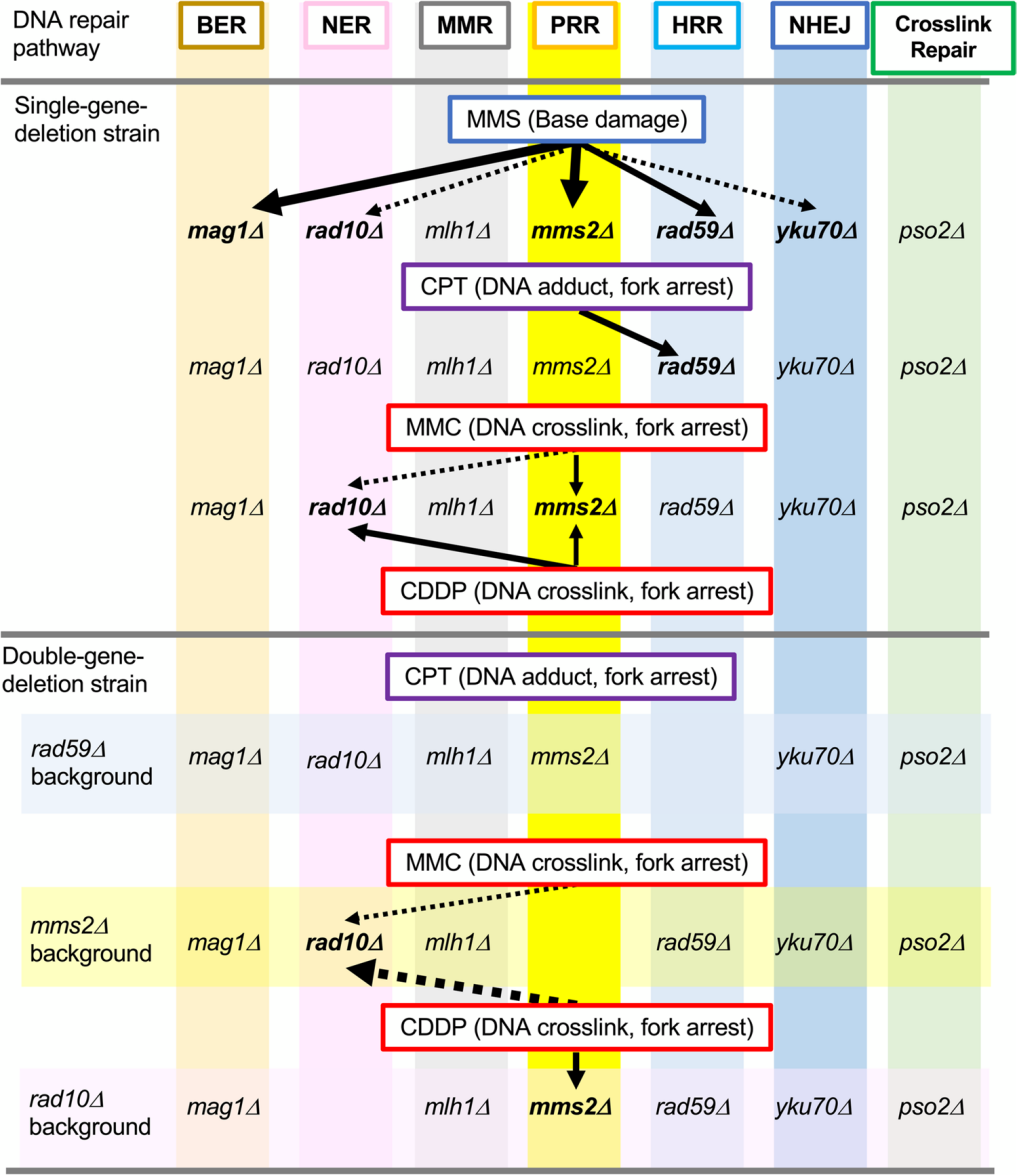
Summary of reporter assays using DNA repair gene-deletion mutants. Results from reporter assays using single-gene-deletion mutants (central part) and double-gene-deletion mutants (bottom part) are summarized. DNA repair pathways and corresponding mutants are also shown (top part and vertical pale-colored columns, respectively). Background alleles (*rad59Δ*, *mms2Δ*, and *rad10Δ*) are indicated (horizontal pale-colored boxes). Genotoxicants used and DNA damage caused (in brackets) are shown for each assay. Strains (in bold) exhibiting highly enhanced responses to the indicated chemical in terms of both luciferase activity and fold induction (bold arrows) and either luciferase activity or fold induction (dashed arrows) are presented (arrow thickness reflects the level of enhancement)

Yeast-reporter assays encounter challenges associated with reduced sensitivity due to diminished reporter response caused by the repair of DNA lesions induced by test chemicals or restricted membrane permeability to chemicals owing to the rigid cell wall. To address this limitation, several studies successfully employed strategies such as the introduction of gene-deletion mutants in DNA repair (Benton et al. 2008; Jia and Xiao 2003; Wei et al. 2013) or cell wall generation and/or transporters (Lichtenberg-Fraté et al. 2003; Walsh et al. 2005; Wei et al. 2013; Zhang et al. 2010, 2008) as host strains. We also tested DNA repair–deficient BY4741 strains, which were generated in a genome-wide yeast gene-deletion project. This was achieved by transfecting a single-copy plasmid containing the secretory *Cypridina* luciferase gene. Notably, we observed markedly higher fold inductions in deletion mutants with *mag1Δ* and *mms2Δ*, *rad59Δ* and *mlh1Δ*, and *mms2Δ* and *mlh1Δ* following exposure to MMS, CPT, and MMC, respectively, compared with those in the wild-type strain (Ochi et al. 2011). In the present study, we systematically generated a series of BY4741-derived single- and double-deletion mutants of DNA repair genes through gene editing. Subsequently, we investigated the response of the luciferase gene in these deletion mutants, all carrying a single-copy reporter plasmid. The summarized results from the reporter assays involving DNA repair gene–deleted mutants are presented in Fig. 8.

Unexpectedly, high fold inductions were not detected in these mutants (Fig. 5) owing to an increase in background luciferase activities in the absence of genotoxic chemicals. This rise can presumably be attributed to the accumulation of endogenous DNA damage resulting from dysfunctional DNA repair processes (Fig. S2). However, significantly elevated reporter activity levels were consistently found in *mag1Δ* and *mms2Δ*, *rad59Δ*, and *mms2Δ* following exposure to MMS, CPT, and MMS, respectively (Fig. 4). In addition, MMS-treated *rad59Δ*, MMC-treated *rad10Δ*, and CDDP-treated *mms2Δ* and *rad10Δ* cells exhibited significantly enhanced activities. In contrast to our previous study, in which MMR-deficient *mlh1Δ* cells displayed high fold inductions upon treatment with CPT and MMC (Ochi et al. 2011), no significant induction of reporter activities was observed in the present study (Fig. 4), although the reasons for this discrepancy remain unclear.

Enhanced luciferase activities and fold inductions induced by MMS were detected in *mag1Δ*, *mms2Δ*, and *rad59Δ* cells, suggesting that BER, PRR, and HRR were involved in repairing DNA lesions caused by the alkylating agent. This observation aligns with a comprehensively screening study of MMS-sensitive mutants (BY4741 background) (Chang et al. 2002), where numerous BER, PRR, and HRR mutants exhibited impaired growth in the presence of 0.035% MMS. Enhanced reporter expression in MMS-treated *mag1Δ* and *mms2Δ* cells was observed in our previous studies (Ochi et al. 2011; Suzuki et al. 2017), consistent with observations from studies involving *mag1Δ* cells (Benton et al. 2008; Jia and Xiao 2003; Wei et al. 2013). Such increased reporter expression is likely caused by the accumulation of MMS-induced DNA damage due to loss of Mag1p DNA glycosylase (Xiao et al. 2001). As the name implies (*methyl methanesulfonate sensitivity-2*), *mms2Δ* cells are hypersensitive to MMS. Additionally, Mms2p forms a complex with Ubc13p, acting as a ubiquitin-conjugating enzyme in error-free PRR (Hofmann and Pickart 1999). Consequently, reporter activity was enhanced in *mms2Δ* cells following MMS treatment. A 7.5-fold higher level of luciferase activity was detected in 1 mM MMS-treated *rad59Δ* cells compared with wild-type cells (Fig. 4a), suggesting the involvement of HRR in repairing MMS-induced DNA damage in yeast.

Despite being representative anticancer drugs, the genotoxicity of the topoisomerase I inhibitor CPT (Khaiwa et al. 2021) and crosslinking agents MMC (Tomasz 1995) and CDDP (Ghosh 2019) was not sensitively detected in our established plasmid-based assays using the wild-type strain (Fig. 2b) (Ochi et al. 2011). Notably, we observed higher levels of luciferase activity under low-concentration CPT exposure in *rad59Δ* cells compared with the wild-type cells (Fig. 4b). This result aligns with our previous findings from a secretary luciferase reporter assay (Ochi et al. 2011), highlighting the crucial role of HRR in DNA damage resulting from CPT-triggered DNA elongation arrests. This is further supported by previous studies showing that mutants with defects in HRR and repair of blocked replication forks are hypersensitive to CPT (Parsons et al. 2004) and that significant growth defects in HRR and DNA damage checkpoint mutants occur in the presence of CPT (Simon et al. 2000).

Higher luciferase activity levels were observed in *mms2Δ* and *rad10Δ* cells compared with BY4741 cells following MMC treatment (Fig. 4c), and luciferase induction due to MMC was higher in *mms2Δ*/*rad10Δ* cells than in *mms2Δ* cells (Fig. 6). Additionally, markedly and moderately enhanced luciferase activities were found in *rad10Δ* and *mms2Δ* cells following CDDP treatment, respectively (Fig. 4d). Notably, enhanced luciferase activity was exclusively found in *mms2Δ*/*rad10Δ* cells among 11 different double-deletion mutants derived from *mms2Δ* and *rad10Δ* strains (Fig. 7). Given that MMC and CDDP are anticancer drugs that cause cytotoxicity primarily by arresting DNA synthesis through crosslinking duplex DNA strands (Tomasz 1995), these observations suggest that PRR and NER play crucial roles in repairing such DNA crosslinks. Grossmann et al*.* showed that three pathways, namely the Pso2p-dependent pathway, Rev3p-dependent PPR, and Rad51p-mediated HRR, are involved in DNA interstrand crosslinks repair, based on hypersensitivity to DNA crosslinkers in the mutants (Grossmann et al. 2001). Growth delay caused by MMC and CDDP was observed in NER, PRR, and HRR yeast mutants but not in BER and MMR mutants (Simon et al. 2000). Genome-wide phenotype analyses using BY4743-derived barcoded homozygous deletion mutants indicated that genes in NER, PRR, and HRR, along with the gene *pso2*, primarily contribute to DNA damage induced by two crosslinkers (Lee et al. 2005; Wu et al. 2004). Another genome-wide study using heterozygous barcode deletion strains showed significant growth defects and lethality in the *pso2Δ* strain and several mutants in NER and HRR in the presence of CDDP (Giaever et al. 2004). Collectively, these studies indicate that four DNA repair pathways (NER, PRR, HRR, and the Pso2p-dependent pathway) contribute to the restoration of DNA damage induced by MMC and CDDP in yeasts, aligning with our observations of enhanced luciferase induction in *rad10Δ* and *mms2Δ* cells treated with MMC and CDDP (Fig. 4c, d). However, the luciferase induction levels in DNA crosslinker–treated *pso2Δ* and *rad59Δ* cells were not significantly high (Fig. 4c, d). Additionally, significantly enhanced luciferase induction was lacking in MMC-treated *pso2Δ/mms2Δ* and *rad59Δ/mms2Δ* cells as well as CDDP-treated *pso2Δ/rad10Δ* and *rad59Δ/rad10Δ* cells (Figs. 6 and 7). Our results suggest that the four DNA repair pathways do not equally contribute to DNA crosslink repair, with NER and PRR predominantly acting in the restoration of DNA damage induced by MMC and CDDP in yeast. Thus, the major contribution of NER and PRR to repair of DNA crosslink repair is further supported by the series of double-gene-deletion mutants in each DNA repair pathway (Fig. 8). He et al. (2021) developed a reporter assay using *GFP*-fused DNA repair genes and successfully assessed DNA damage types based on the expression profiles after chemical exposure. This may be a useful alternative for determining DNA repair pathway involved in chemical-induced DNA lesions, including those induced by anticancer drugs, and reporter assays involving DNA repair–deficient strains may hold utility for screening lead compounds for novel anticancer drugs.

Elevated background levels of luciferase activity were observed in some DNA repair gene-deletion mutants, such as *mag1Δ*, *rad10Δ*, *mms2Δ*, and *rad59Δ* strains, without chemical treatment, whereas *pso2Δ*, *mlh1Δ*, and *yku70Δ* strains did not exhibit such increases (Fig. S2). These observations can be attributed to increased endogenous DNA damage resulting from functional defects in the corresponding DNA repair gene. In particular, consistently high levels of luciferase induction in *mag1Δ* and *rad10Δ* cells were noted, suggesting that endogenously generated DNA damage, such as oxidative DNA lesions caused by respiration, is primarily repaired by excision DNA repair. Despite marked luciferase induction in the presence of genotoxic chemicals, the high background luciferase activity levels in several DNA repair–deficient mutants led to decreases in fold induction (Figs. 4 and 5). In the previous study, using a reporter plasmid with the ^P^*RNR3*-linked *Cypridina* luciferase gene, we demonstrated significantly high fold inductions in mutant strains with *mms2Δ* and *mlh1Δ*, *rad59Δ* and *mlh1Δ*, and *mms2Δ* and *mlh1Δ* following exposure to MMS, CPT, and MMC, respectively (Ochi et al. 2011). These results are consistent with our present observations, except for those in *mlh1Δ* cells. Notably, in our previous study, we did not observe enhanced secretory luciferase background activities in DNA repair–deficient mutants in the absence of DNA-damaging agents, which could account for the discrepant results in fold induction observed in our two studies.

Finally, we tested three gene-deletion strains with defects in cell permeability and chemical transport to increase the effective concentrations of tested agents in yeast cells. Although two reporter strains with *PDR1-* and *PDR3-*deleted alleles exhibited a reporter gene response comparable to that of wild-type BY4741 cells following exposure to HU, the *erg6Δ* reporter strain showed enhanced luciferase induction (Fig. S4). Limited increases in ^P^*RNR3*-mediated reporter induction were observed following exposure to four genotoxic chemicals in a prior study (Zhang et al. 2008), although *ERG6* inactivation is known to alter membrane permeability, increasing yeast’s sensitivity to chemicals (Emter et al. 2002; Welihinda et al. 1994). Hence, employing gene editing to introduce multiple deletions in DNA repair genes and cell permeability–related genes enabled the development of yeast strains tailored for a reporter assay with enhanced sensitivity to genotoxic chemicals.

In conclusion, we developed three yeast-based luciferase reporter assays tailored for genotoxicity testing, each demonstrating distinct luciferase induction upon exposure to representative genotoxic chemicals, measuring both luciferase activity and fold induction. Employing the CRISPR/Cas9-mediated gene editing technique, we also facilitated the preparation of chromosomally integrated reporter strains and genetically modified host strains. Using this approach, we generated 10 single- and 11 double-deletion mutants, successfully showing that NER and PRR mainly play crucial roles in repairing DNA strand crosslinks in BY4741, as evidenced by mutagen-dependent enhanced induction of luciferase activity. Consequently, this study will be useful for developing improved yeast-based genotoxicity tests and tools to further investigate DNA repair mechanisms and screen potential anticancer compounds.

## Supplementary Information

Below is the link to the electronic supplementary material.

Supplementary file1 (PDF 1.03 MB)

## Data Availability

The datasets generated and/or analyzed during the current study are available from the corresponding author on reasonable request.

## References

[CR1] Afanassiev V, Sefton M, Anantachaiyong T, Barker G, Walmsley R, Wölfl S (2000) Application of yeast cells transformed with GFP expression constructs containing the *RAD54* or *RNR2* promoter as a test for the genotoxic potential of chemical substances. Mutat Res 464:297–308. 10.1016/s1383-5718(99)00209-0 10648917 10.1016/s1383-5718(99)00209-0

[CR2] Ames BN, Durston WE, Yamasaki E, Lee FD (1973) Carcinogens are mutagens: a simple test system combining liver homogenates for activation and bacteria for detection. Proc Natl Acad Sci U S A 70:2281-2285. 10.1073/pnas.70.8.2281 10.1073/pnas.70.8.2281 PMC433718 4151811

[CR3] Benton MG, Glasser NR, Palecek SP (2007) The utilization of a *Saccharomyces**cerevisiae**HUG1P-GFP* promoter-reporter construct for the selective detection of DNA damage. Mutat Res 633:21–34. 10.1016/j.mrgentox.2007.05.002 17618162 10.1016/j.mrgentox.2007.05.002

[CR4] Benton MG, Glasser NR, Palecek SP (2008) Deletion of *MAG1* and *MRE11* enhances the sensitivity of the *Saccharomyces**cerevisiae HUG1P*-GFP promoter-reporter construct to genotoxicity. Biosens Bioelectron 24:736–741. 10.1016/j.bios.2008.06.033 18693109 10.1016/j.bios.2008.06.033 PMC4526160

[CR5] Biegel JA, Leslie DS, Bigner DD, Bigner SH (1987) Hydroxyurea synchronization increases mitotic yield in human glioma cell lines. Acta Neuropathol 73:309–312. 10.1007/BF00686628 3618122 10.1007/BF00686628

[CR6] Billinton N, Barker MG, Michel CE, Knight AW, Heyer WD, Goddard NJ, Fielden PR, Walmsley RM (1998) Development of a green fluorescent protein reporter for a yeast genotoxicity biosensor. Biosens Bioelectron 13:831–838. 10.1016/s0956-5663(98)00049-9 9828379 10.1016/s0956-5663(98)00049-9

[CR7] Boronat S, Pina B (2006) Development of *RNR3-* and *RAD54*-GUS reporters for testing genotoxicity in *Saccharomyces**cerevisiae*. Anal Bioanal Chem 386:1625–1632. 10.1007/s00216-006-0751-4 17004060 10.1007/s00216-006-0751-4

[CR8] Bui VN, Nguyen TT, Bettarel Y, Nguyen TH, Pham TL, Hoang TY, Nguyen VT, Nghiem NM, Wolfl S (2015) Genotoxicity of chemical compounds identification and assessment by yeast cells transformed with GFP reporter constructs regulated by the *PLM2* or *DIN7* promoter. Int J Toxicol 34:31–43. 10.1177/1091581814566870 25691521 10.1177/1091581814566870

[CR9] Bui VN, Nguyen TT, Mai CT, Bettarel Y, Hoang TY, Trinh TT, Truong NH, Chu HH, Nguyen VT, Nguyen HD, Wölfl S (2016) Procarcinogens - determination and evaluation by yeast-based biosensor transformed with plasmids incorporating *RAD54* reporter construct and cytochrome *P450* genes. PLoS One 11:e0168721. 10.1371/journal.pone.0168721 28006013 10.1371/journal.pone.0168721 PMC5179006

[CR10] Cahill PA, Knight AW, Billinton N, Barker MG, Walsh L, Keenan PO, Williams CV, Tweats DJ, Walmsley RM (2004) The GreenScreen genotoxicity assay: a screening validation programme. Mutagenesis 19:105–119. 10.1093/mutage/geh015 14981157 10.1093/mutage/geh015

[CR11] Chang M, Bellaoui M, Boone C, Brown GW (2002) A genome-wide screen for methyl methanesulfonate-sensitive mutants reveals genes required for S phase progression in the presence of DNA damage. Proc Natl Acad Sci U S A 99:16934-16939. 10.1073/pnas.262669299 10.1073/pnas.262669299 PMC139247 12482937

[CR12] Dunham M, Gartenberg MR, Brown GW (2015) Methods in yeast genetics and genomics. Cold Spring Harbor Laboratory Press, New York

[CR13] Eki T (2018) Yeast-based genotoxicity tests for assessing DNA alterations and DNA stress responses: a 40-year overview. Appl Microbiol Biotechnol 102:2493–2507. 10.1007/s00253-018-8783-1 29423630 10.1007/s00253-018-8783-1

[CR14] Elledge SJ, Zhou Z, Allen JB, Navas TA (1993) DNA damage and cell cycle regulation of ribonucleotide reductase. BioEssays 15:333–339. 10.1002/bies.950150507 8343143 10.1002/bies.950150507

[CR15] Emter R, Heese-Peck A, Kralli A (2002) *ERG6* and *PDR5* regulate small lipophilic drug accumulation in yeast cells via distinct mechanisms. FEBS Lett 521:57–61. 10.1016/s0014-5793(02)02818-1 12067726 10.1016/s0014-5793(02)02818-1

[CR16] Endo-Ichikawa Y, Kohno H, Tokunaga R, Taketani S (1995) Induction in the gene *RNR3* in *Saccharomyces**cerevisiae* upon exposure to different agents related to carcinogenesis. Biochem Pharmacol 50:1695–1699. 10.1016/0006-2952(95)02071-3 7503773 10.1016/0006-2952(95)02071-3

[CR17] Friedberg EC, Walker GC, Siede W, Wood RD, Schultz RA, Ellenberger T (2005) DNA repair and mutagenesis, 2nd edn. American Society for Microbiology Press, Washington, DC

[CR18] Gao CY, Pinkham JL (2000) Tightly regulated, β-estradiol dose-dependent expression system for yeast. Biotechniques 29:1226–1231. 10.2144/00296st02 11126125 10.2144/00296st02

[CR19] Ghosh S (2019) Cisplatin: the first metal based anticancer drug. Bioorg Chem 88:102925. 10.1016/j.bioorg.2019.102925 31003078 10.1016/j.bioorg.2019.102925

[CR20] Giaever G, Flaherty P, Kumm J, Proctor M, Nislow C, Jaramillo DF, Chu AM, Jordan MI, Arkin AP, Davis RW (2004) Chemogenomic profiling: identifying the functional interactions of small molecules in yeast. Proc Natl Acad Sci U S A 101:793-798. 10.1073/pnas.0307490100 10.1073/pnas.0307490100 PMC321760 14718668

[CR21] Gietz D, St Jean A, Woods RA, Schiestl RH (1992) Improved method for high efficiency transformation of intact yeast cells. Nucleic Acids Res 20:1425. 10.1093/nar/20.6.1425 1561104 10.1093/nar/20.6.1425 PMC312198

[CR22] Grossmann KF, Ward AM, Matkovic ME, Folias AE, Moses RE (2001) *S.**cerevisiae* has three pathways for DNA interstrand crosslink repair. Mutat Res 487:73–83. 10.1016/s0921-8777(01)00106-9 11738934 10.1016/s0921-8777(01)00106-9

[CR23] He Y, Ding H, Xia X, Qi W, Wang H, Liu W, Zheng F (2021) GFP-fused yeast cells as whole-cell biosensors for genotoxicity evaluation of nitrosamines. Appl Microbiol Biotechnol 105:5607–5616. 10.1007/s00253-021-11426-4 34228183 10.1007/s00253-021-11426-4

[CR24] Hofmann RM, Pickart CM (1999) Noncanonical *MMS2*-encoded ubiquitin-conjugating enzyme functions in assembly of novel polyubiquitin chains for DNA repair. Cell 96:645–653. 10.1016/s0092-8674(00)80575-9 10089880 10.1016/s0092-8674(00)80575-9

[CR25] Ichikawa K, Eki T (2006) A novel yeast-based reporter assay system for the sensitive detection of genotoxic agents mediated by a DNA damage-inducible LexA-GAL4 protein. J Biochem 139:105–112. 10.1093/jb/mvj011 16428325 10.1093/jb/mvj011

[CR26] Jia X, Xiao W (2003) Compromised DNA repair enhances sensitivity of the yeast *RNR3-lacZ* genotoxicity testing system. Toxicol Sci 75:82–88. 10.1093/toxsci/kfg158 12805645 10.1093/toxsci/kfg158

[CR27] Jia X, Zhu Y, Xiao W (2002) A stable and sensitive genotoxic testing system based on DNA damage induced gene expression in *Saccharomyces**cerevisiae*. Mutat Res 519:83–92. 10.1016/s1383-5718(02)00129-8 12160894 10.1016/s1383-5718(02)00129-8

[CR28] Khaiwa N, Maarouf NR, Darwish MH, Alhamad DWM, Sebastian A, Hamad M, Omar HA, Orive G, Al-Tel TH (2021) Camptothecin’s journey from discovery to WHO Essential Medicine: fifty years of promise. Eur J Med Chem 223:113639. 10.1016/j.ejmech.2021.113639 34175539 10.1016/j.ejmech.2021.113639

[CR29] Knight AW, Billinton N, Cahill PA, Scott A, Harvey JS, Roberts KJ, Tweats DJ, Keenan PO, Walmsley RM (2007) An analysis of results from 305 compounds tested with the yeast *RAD54-GFP* genotoxicity assay (GreenScreen GC)-including relative predictivity of regulatory tests and rodent carcinogenesis and performance with autofluorescent and coloured compounds. Mutagenesis 22:409–416. 10.1093/mutage/gem036 17906314 10.1093/mutage/gem036

[CR30] Knight AW, Goddard NJ, Billinton N, Cahill PA, Walmsley RM (2002) Fluorescence polarization discriminates green fluorescent protein from interfering autofluorescence in a microplate assay for genotoxicity. J Biochem Biophys Methods 51:165–177. 10.1016/s0165-022x(02)00014-3 12062116 10.1016/s0165-022x(02)00014-3

[CR31] Lee W, St Onge RP, Proctor M, Flaherty P, Jordan MI, Arkin AP, Davis RW, Nislow C, Giaever G (2005) Genome-wide requirements for resistance to functionally distinct DNA-damaging agents. PLoS Genet 1:e24. 10.1371/journal.pgen.0010024 16121259 10.1371/journal.pgen.0010024 PMC1189734

[CR32] Lees ND, Skaggs B, Kirsch DR, Bard M (1995) Cloning of the late genes in the ergosterol biosynthetic pathway of Saccharomyces cerevisiae–a review. Lipids 30:221–226. 10.1007/BF02537824 7791529 10.1007/BF02537824

[CR33] Lichtenberg-Fraté H, Schmitt M, Gellert G, Ludwig J (2003) A yeast-based method for the detection of cyto and genotoxicity. Toxicol in Vitro 17:709–716. 10.1016/s0887-2333(03)00129-2 14599467 10.1016/s0887-2333(03)00129-2

[CR34] Liu X, Kramer JA, Swaffield JC, Hu Y, Chai G, Wilson AG (2008) Development of a highthroughput yeast-based assay for detection of metabolically activated genotoxins. Mutat Res 653:63–69. 10.1016/j.mrgentox.2008.03.006 18485802 10.1016/j.mrgentox.2008.03.006

[CR35] Lu Y, Tian Y, Wang R, Wu Q, Zhang Y, Li X (2015) Dual fluorescent protein-based bioassay system for the detection of genotoxic chemical substances in *Saccharomyces**cerevisiae*. Toxicol Mech Methods 25:698–707. 10.3109/15376516.2015.1070305 26228088 10.3109/15376516.2015.1070305

[CR36] Mamnun YM, Pandjaitan R, Mahé Y, Delahodde A, Kuchler K (2002) The yeast zinc finger regulators Pdr1p and Pdr3p control pleiotropic drug resistance (PDR) as homo- and heterodimers *in**vivo*. Mol Microbiol 46:1429–1440. 10.1046/j.1365-2958.2002.03262.x 12453227 10.1046/j.1365-2958.2002.03262.x

[CR37] Mans R, van Rossum HM, Wijsman M, Backx A, Kuijpers NG, van den Broek M, Daran-Lapujade P, Pronk JT, van Maris AJ, Daran JM (2015) CRISPR/Cas9: a molecular Swiss army knife for simultaneous introduction of multiple genetic modifications in *Saccharomyces**cerevisiae*. FEMS Yeast Res 15:fov004. 10.1093/femsyr/fov004 25743786 10.1093/femsyr/fov004 PMC4399441

[CR38] Masser AE, Kandasamy G, Kaimal JM, Andréasson C (2016) Luciferase NanoLuc as a reporter for gene expression and protein levels in *Saccharomyces**cerevisiae*. Yeast 33:191–200. 10.1002/yea.3155 26860732 10.1002/yea.3155 PMC5069653

[CR39] Ochi Y, Sugawara H, Iwami M, Tanaka M, Eki T (2011) Sensitive detection of chemical-induced genotoxicity by the *Cypridina* secretory luciferase reporter assay, using DNA repair-deficient strains of *Saccharomyces**cerevisiae*. Yeast 28:265–278. 10.1002/yea.1837 21456053 10.1002/yea.1837

[CR40] Parsons AB, Brost RL, Ding H, Li Z, Zhang C, Sheikh B, Brown GW, Kane PM, Hughes TR, Boone C (2004) Integration of chemical-genetic and genetic interaction data links bioactive compounds to cellular target pathways. Nat Biotechnol 22:62–69. 10.1038/nbt919 14661025 10.1038/nbt919

[CR41] Rosebrock AP (2016) Methods for synchronization and analysis of the budding yeast cell cycle. In: Andrews B, Boone C, Davis TN, Fields S (eds) Budding yeast A laboratory manual. Cold Spring Harbor Laboratory Press, Cold Spring Harbor, Nwe York, pp 239–263 10.1101/pdb.top080630 28049810

[CR42] Shichinohe M, Ohkawa S, Hirose Y, Eki T (2023) Sensing chemical-induced genotoxicity and oxidative stress via yeast-based reporter assays using NanoLuc luciferase. PLoS One 18:e0294571. 10.1371/journal.pone.0294571 37992069 10.1371/journal.pone.0294571 PMC10664910

[CR43] Simon JA, Szankasi P, Nguyen DK, Ludlow C, Dunstan HM, Roberts CJ, Jensen EL, Hartwell LH, Friend SH (2000) Differential toxicities of anticancer agents among DNA repair and checkpoint mutants of *Saccharomyces**cerevisiae*. Cancer Res 60:328–333 10667584

[CR44] Suzuki H, Sakabe T, Hirose Y, Eki T (2017) Development and evaluation of yeast-based GFP and luciferase reporter assays for chemical-induced genotoxicity and oxidative damage. Appl Microbiol Biotechnol 101:659–671. 10.1007/s00253-016-7911-z 27766356 10.1007/s00253-016-7911-z

[CR45] Symington LS (2002) Role of *RAD52* epistasis group genes in homologous recombination and double-strand break repair. Microbiol Mol Biol Rev 66:630–670. 10.1128/MMBR.66.4.630-670.2002 12456786 10.1128/MMBR.66.4.630-670.2002 PMC134659

[CR46] Tomasz M (1995) Mitomycin C: small, fast and deadly (but very selective). Chem Biol 2:575–579. 10.1016/1074-5521(95)90120-5 9383461 10.1016/1074-5521(95)90120-5

[CR47] Van Gompel J, Woestenborghs F, Beerens D, Mackie C, Cahill PA, Knight AW, Billinton N, Tweats DJ, Walmsley RM (2005) An assessment of the utility of the yeast GreenScreen assay in pharmaceutical screening. Mutagenesis 20:449–454. 10.1093/mutage/gei062 16291732 10.1093/mutage/gei062

[CR48] Walmsley RM, Billinton N, Heyer WD (1997) Green fluorescent protein as a reporter for the DNA damage-induced gene *RAD54* in *Saccharomyces**cerevisiae*. Yeast 13:1535–1545. 10.1002/(SICI)1097-0061(199712)13:16%3c1535::AID-YEA221%3e3.0.CO;2-2 9509573 10.1002/(SICI)1097-0061(199712)13:16<1535::AID-YEA221>3.0.CO;2-2

[CR49] Walsh L, Hastwell PW, Keenan PO, Knight AW, Billinton N, Walmsley RM (2005) Genetic modification and variations in solvent increase the sensitivity of the yeast *RAD54*-GFP genotoxicity assay. Mutagenesis 20:317–327. 10.1093/mutage/gei044 15985442 10.1093/mutage/gei044

[CR50] Wei T, Zhang C, Xu X, Hanna M, Zhang X, Wang Y, Dai H, Xiao W (2013) Construction and evaluation of two biosensors based on yeast transcriptional response to genotoxic chemicals. Biosens Bioelectron 44:138–145. 10.1016/j.bios.2013.01.029 23416315 10.1016/j.bios.2013.01.029

[CR51] Welihinda AA, Beavis AD, Trumbly RJ (1994) Mutations in *LIS1* (*ERG6*) gene confer increased sodium and lithium uptake in *Saccharomyces**cerevisiae*. Biochim Biophys Acta 1193:107–117. 10.1016/0005-2736(94)90339-5 8038180 10.1016/0005-2736(94)90339-5

[CR52] Westerink WM, Stevenson JC, Lauwers A, Griffioen G, Horbach GJ, Schoonen WG (2009) Evaluation of the Vitotox and RadarScreen assays for the rapid assessment of genotoxicity in the early research phase of drug development. Mutat Res 676:113–130. 10.1016/j.mrgentox.2009.04.008 19393335 10.1016/j.mrgentox.2009.04.008

[CR53] Wu HI, Brown JA, Dorie MJ, Lazzeroni L, Brown JM (2004) Genome-wide identification of genes conferring resistance to the anticancer agents cisplatin, oxaliplatin, and mitomycin C. Cancer Res 64:3940–3948. 10.1158/0008-5472.CAN-03-3113 15173006 10.1158/0008-5472.CAN-03-3113

[CR54] Xiao W, Chow BL, Hanna M, Doetsch PW (2001) Deletion of the *MAG1* DNA glycosylase gene suppresses alkylation-induced killing and mutagenesis in yeast cells lacking AP endonucleases. Mutat Res 487:137–147. 10.1016/s0921-8777(01)00113-6 11738940 10.1016/s0921-8777(01)00113-6

[CR55] Zhang M, Hanna M, Li J, Butcher S, Dai H, Xiao W (2010) Creation of a hyperpermeable yeast strain to genotoxic agents through combined inactivation of *PDR* and *CWP* genes. Toxicol Sci 113:401–411. 10.1093/toxsci/kfp267 19884123 10.1093/toxsci/kfp267

[CR56] Zhang M, Liang Y, Zhang X, Xu Y, Dai H, Xiao W (2008) Deletion of yeast *CWP* genes enhances cell permeability to genotoxic agents. Toxicol Sci 103:68–76. 10.1093/toxsci/kfn034 18281714 10.1093/toxsci/kfn034

[CR57] Zhang M, Zhang C, Li J, Hanna M, Zhang X, Dai H, Xiao W (2011) Inactivation of *YAP1* enhances sensitivity of the yeast *RNR3-lacZ* genotoxicity testing system to a broad range of DNA-damaging agents. Toxicol Sci 120:310–321. 10.1093/toxsci/kfq391 21205635 10.1093/toxsci/kfq391

[CR58] Zhou Z, Elledge SJ (1992) Isolation of *crt* mutants constitutive for transcription of the DNA damage inducible gene *RNR3* in *Saccharomyces**cerevisiae*. Genetics 131:851–866. 10.1093/genetics/131.4.851 1516817 10.1093/genetics/131.4.851 PMC1205097

