# Supplementary material for: Sensing chemical-induced DNA damage using CRISPR/Cas9-mediated gene-deletion yeast-reporter strains
Source: Appl Microbiol Biotechnol. 2024 Feb 1;108(1):188. doi: 10.1007/s00253-024-13020-w (PMC10834598; doi:10.1007/s00253-024-13020-w)
Supplement: Supplementary file 1 — Supplementary file1 (PDF 1.03 MB) [file 253_2024_13020_MOESM1_ESM.pdf]

Sensing chemical-induced DNA damage using CRISPR/Cas9-mediated gene-deletion yeast-reporter strains

Kosuke Yamamoto[1], Shintaro Tochikawa[1], Yuuki Miura[1], Shogo Matsunobu[1], Yuu Hirose[1, 2], and Toshihiko Eki[1]*

[1]Molecular Genetics Laboratory, and [2]Laboratory of Genomics and Photobiology, Department of Applied Chemistry and Life Science, Toyohashi University of Technology, 1-1 Hibarigaoka, Tempaku-cho, Toyohashi, Aichi 441-8580, Japan

*Corresponding author. Toshihiko Eki, Molecular Genetics Laboratory, Department of Applied Chemistry and Life Science, Toyohashi University of Technology, 1-1 Hibarigaoka, Tempaku-cho, Toyohashi, Aichi 441-8580, Japan. E-mail: eki@chem.tut.ac.jp; Tel: +81-532-44-6907; Fax: +81-532-44-6929

Fig. S1. Luciferase induction with five genotoxic chemicals in the BY4741 strain carrying a chromosomally integrated and a multicopy plasmid with a *RNR3* promoter-driven luciferase gene.

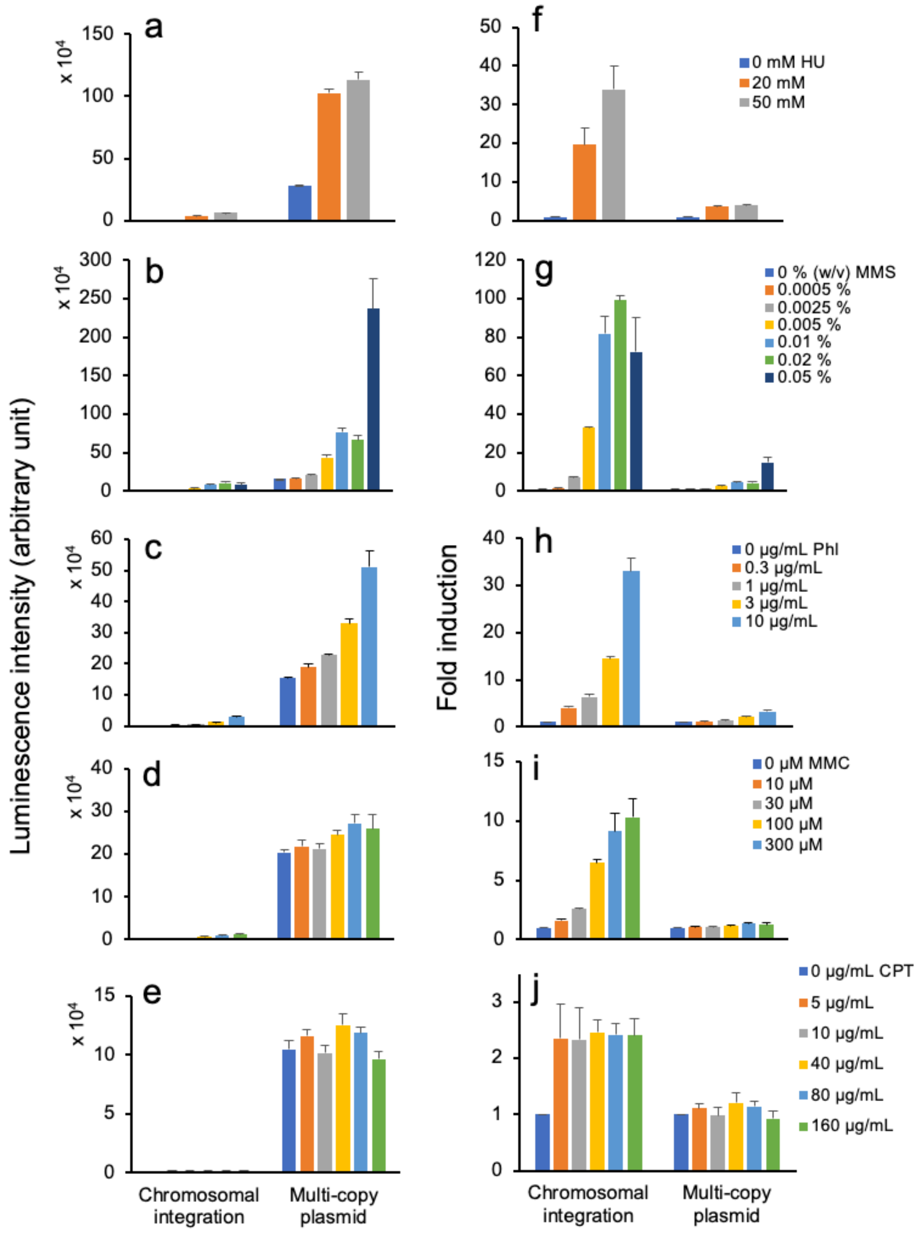

Yeast cells with a chromosomally integrated ^{P}*RNR3*-luciferase gene (*luc2*) at the *CAN1* locus (left in each panel) and plasmid pESC-HIS*ΔGAL1/10*-^{P}*RNR3-luc2* (right in each panel) were cultured for 8 h in the absence or presence of the indicated concentrations of hydroxyurea (HU) (**a, f**), methyl methanesulfonate (MMS) (**b, g**), phleomycin (Phl) (**c, h**), mitomycin C (MMC) (**d, i**), and camptothecin (CPT) (**e, j**). Luminescence intensity derived from luciferase activity (**a–e**) and fold induction (**f–j**) are shown with standard deviations.

Fig. S2. Level of luciferase activity without chemicals in the wild-type and seven DNA repair gene-deleted strains carrying a single-copy *RNR3* promoter-driven luciferase reporter plasmid.

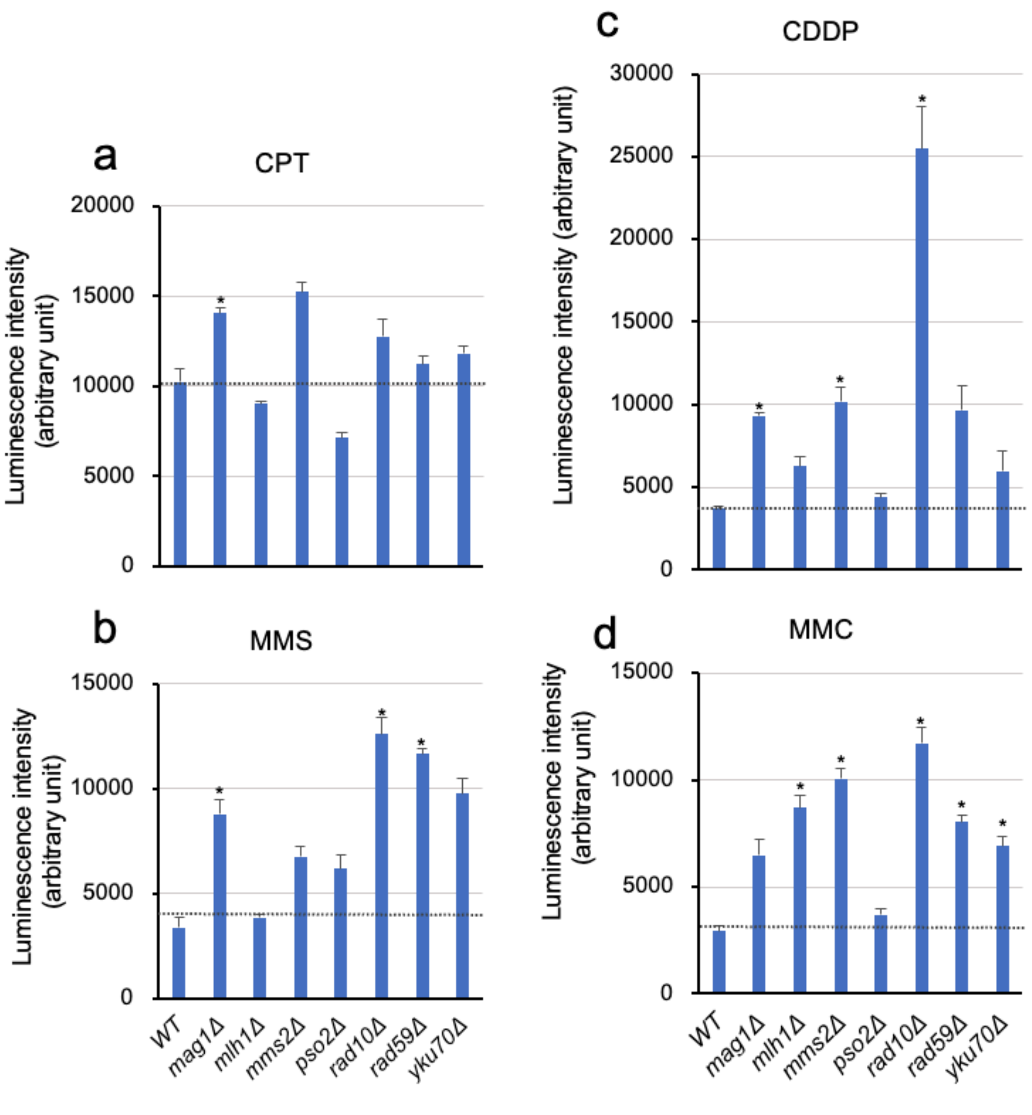

The yeast cells indicated containing plasmid pRS313-HIS3-^{P}*RNR3-luc2* were cultured for 8 h without CPT (**a**), MMS (**b**), CDDP (**c**), or MMC (**d**) in each experiment. Chemiluminescence intensity in the cells was determined and is shown with standard deviations. Bar plots with asterisks indicate the mutant cells showed significant luminescence intensity compared with that of the corresponding wild-type cells (Student's *t*-test, $p < 0.01$; Table S6).

Fig. S3. Luciferase induction with CPT in yeast DNA repair double-gene deletion mutants with *rad59Δ* allele.

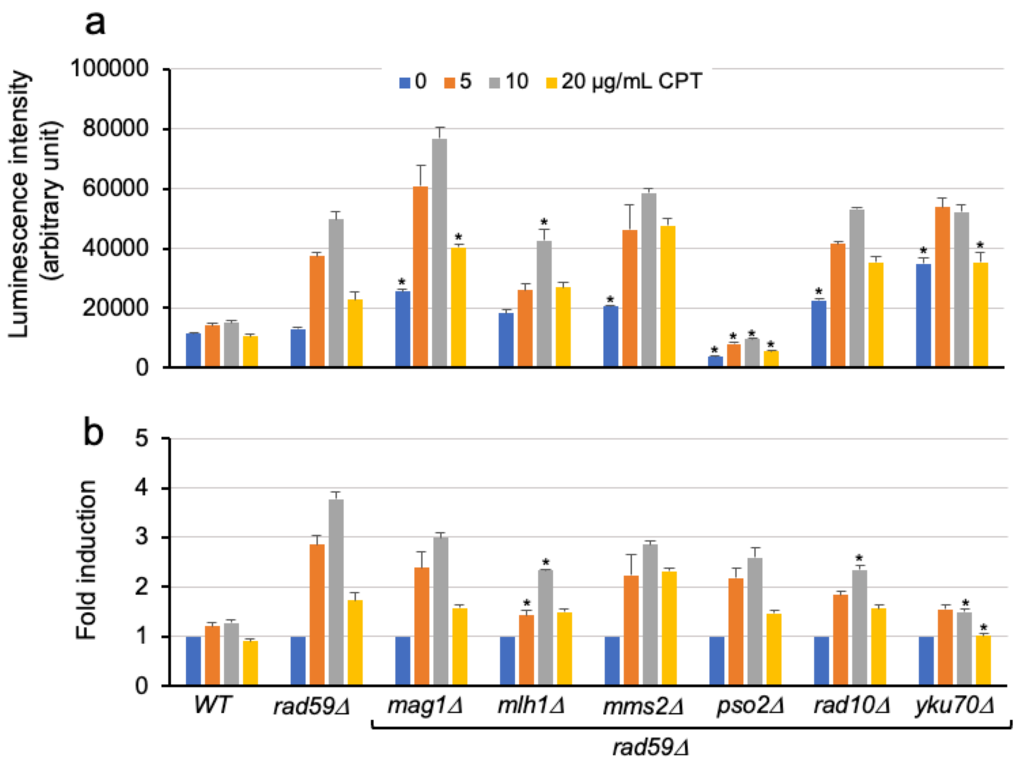

Wild-type BY4741, *rad59Δ*, and six *rad59Δ*-derived double-gene deletion mutants with plasmid pRS313-HIS3-^{P}*RNR3-luc2* were cultured with or without indicated concentrations of CPT for 8 h. The luciferase activity (**a**) and fold induction (**b**) in the cells are shown with standard deviations. Bar plots with asterisks indicate the double-gene deletion mutant cells show significant luminescent intensity compared with that in the corresponding *rad59Δ* cells (Student's *t*-test, $p < 0.01$; Table S7).

Fig. S4. Luciferase induction with HU in three gene-deletion mutants defective in cell permeability.

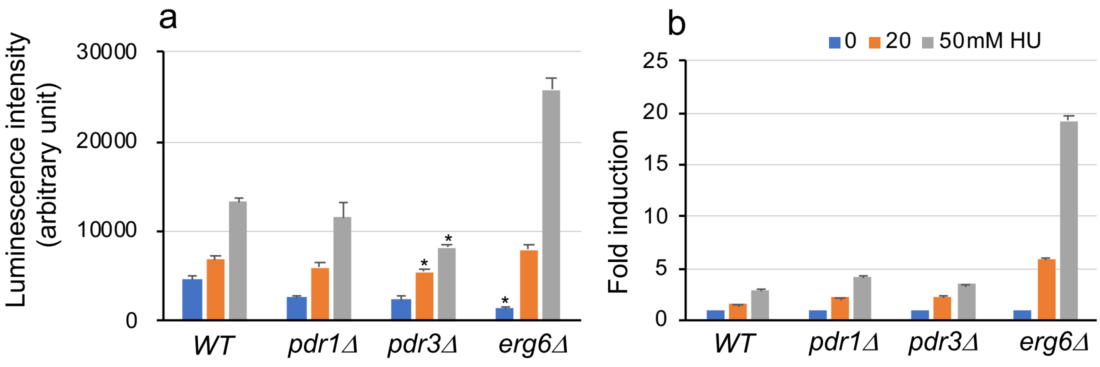

Wild-type BY4741, *pdr1Δ*, *pdr3Δ*, and *erg6Δ* cells were cultured with or without indicated concentrations of HU for 8 h. The luciferase activity (**a**) and fold induction (**b**) in cells are shown with standard deviations. Bar plots with asterisks indicate the gene-deletion mutant cells with significant luminescent intensity compared with that of the corresponding wild-type cells (Student's *t*-test, $p < 0.01$; Table S10).

Table S1. PCR primers and nucleotide sequences used in this study

Primer	Nucleotide sequence (5' to 3')	Use
pRS313-*Not*-invR	CCGCGGTGGAGCTCCAATTCGCCCTA	Preparation of pRS313-*HIS3*-^{P}*RNR3-luc2* plasmid
pRS313-*Bam*-invF	CCCGGGCTGCAGGAATTCGATATCAAG	
pRS313-*Not*-TRX2yNlucP-IFF	GGAGCTCCACCGCGGCGCTATTACGCCAGCTGAATTGGAGC	
pRS313-*Bam*-TRX2yNlucP-IFR	TTCCTGCAGCCCGGGTAATGCAGCTGGATCTTCGAGCGTCC	
TADH1-5F2-25CAN1-Rtail	CAGTTGTCTCTATCAATGAAAATTTGCGTCCATTCGCCATTCAGGCTGCG	Preparation of a reporter DNA for chromosomal integration
TCYC1-3R3-25CAN1-Ftail	GATCAAAGGTAATAAAACGTCATATTACCGCTCGCCGCAGCCGAACGACC	
5'-CAN1-F	AATAGGGCGAACTTGAAGAATAACC	
5-CAN1-R_5F2_30tail	AGTTGCGCAGCCTGAATGGCGAATGGACGCAAATTTTCATTGATAGAGACAACTG	
3-CAN1-F_3R3_30tail	CGCTCGGTCGGCTGCGGCGAGCGGTAATATGACGTTTTATTACCTTTGATC	
3'-CAN1-R	TACTTGAAGGTCTGAAGGAGTTTCA	
CAN1-5check-F	TATCTTTAACAGATTCCAAACCCTA	Confirmation of chromosomal integration by colony PCR
CAN1orf-R	GGGAAAGAGCGCAATGGATACAATTC	
CAN1orf-F	ATTTAAAGCTAAATTAATGCCCGG	
CAN1_dg rv	GGTTCTAGGTTCGGGTGACG	
luc2-SQR2	ACGAATACGACGGTGGGCTGGC	
p414-2873dIFF	GAATTCCTGCAGCCCGGGAAGCTGGAGCTCATAGCTTCAAAATG	Preparation of Cas9 expression plasmid
p414-4653dIFR	ACTAGTGGATCCCCCGGGCCGCAAATTAAAGCCTTCGAGCGTC	
#6005_p426CRISPR rv2	TTTATCTTTCACTGCGGAGAAGTTTCG	Preparation of gRNA expression plasmid
MAG1_#6006_p426CR fw2	TGATCTTAAGCATAAGAAATACAACGTTTTAGAGCTAGAAATAGCAAGTTAAAATAAG	
MLH1_#6006_p426CR fw2	TGATCGGCTTTTCTTGTAAAAAGTTGTTTTAGAGCTAGAAATAGCAAGTTAAAATAAG	
MMS2_#6006_p426CR fw2	TGATCCTGTTTTCATGATTACTATGGTTTTAGAGCTAGAAATAGCAAGTTAAAATAAG	
PSO2_#6006_p426CR fw2	TGATCTGCAATAATAATGCTTTTCCGTTTTAGAGCTAGAAATAGCAAGTTAAAATAAG	
RAD10_#6006_p426CR fw2	TGATCTACTTCATTTGAAAGTATATGTTTTAGAGCTAGAAATAGCAAGTTAAAATAAG	
RAD59_#6006_p426CR fw2	TGATCTTTACCACAATAATAAATATGTTTTAGAGCTAGAAATAGCAAGTTAAAATAAG	
YKU70_#6006_p426CR fw2	TGATCGAGAAAACAAATTTTATAGTGTTTTAGAGCTAGAAATAGCAAGTTAAAATAAG	
PDR1_#6006_p426CR fw2	TGATCTAATAAGATTTTGTTCATATGTTTTAGAGCTAGAAATAGCAAGTTAAAATAAG	
PDR3_#6006_p426CR fw2	TGATCTTTTAAAAATATGATTCTTAGTTTTAGAGCTAGAAATAGCAAGTTAAAATAAG	
ERG6_#6006_p426CR fw2	TGATCAGATTTCTATGAATATGGTTGTTTTAGAGCTAGAAATAGCAAGTTAAAATAAG	
MAG1-5-233F	GTTAAGTATACTTTCTTATTCGACC	Preparation of repair gene fragment
MAG1-5-233R-tail	GTCAGTAATACATGATATCTTTAAGTGAGACCAATCCCTCTCTAGCTTCTC	
MAG1-3-222F	CTTAAAGATATCATGTATTACTGA	
MAG1-3-222R	TAAAGGTATTCTTATGAGAATTGAC	
MLH1-5-206F	CAAAGATAGTGTAGGAGGCGCTGCT	
MLH1-5-206R-tail	GGTATTACAGCCAAAACGTTTTAAAGTATTGATAGGTCTATGTTATTTTTACC	
MLH1-3-217F	CTTTAAAACGTTTTGGCTGTAATAC	
MLH1-3-217R	AACAATACGGAACCAAGTGACTTCAG	
MMS2-5-172F	AGGAATTATTCCTAGGGTAACACGC	
MMS2-5-172R-tail	ATACTGTTTAGGAAAAAGTAGATAATTTTGTGTAAACGCTGCTTTCTTCTAC	
MMS2-3-237F	TTATCTACTTTTTCCTAAACAGTAT	
MMS2-3-237R	GATCTAAGGGTTTACCTTCGTGTGC	
PSO2-5-207F	TACTTAGTAGTCCCATGTATTCTTC	
PSO2-5-207R-tail	ACGTACGTACATCTTACATAACACAAATCAACCGACCACCCAAATTAGTCAC	
PSO2-3-175F	TGTGTTATGTAAGATGTACGTACGT	
PSO2-3-175R	CTATTGATTTAGCCAACGATCGTAG	
RAD10-5-182F	CATACTTTTTTGAGAGGACATGGCTTG	
RAD10-5-182R-tail	GATTTATTAAAAGAAAATAGGAATTGTCTTCTAGGATAACCAACTTTGTTTATG	
RAD10-3-201F	ACAATTCCTATTTTCTTTTAATAAATC	
RAD10-3-201R	ATAAAGTTATCTACAGCAGAAATTCCC	
RAD59-5-215F	TCAAGTTGGTGTCACGTCACGTGCT	
RAD59-5-215R-tail	CAAGCAAAATAAATTTGCTACTTGTGCTATTTTGTCTGTTTATCCTGAAATATG	
RAD59-3-251F	GCACAAGTAGCAAATTTATTTTGCTTG	
RAD59-3-251R	TTACCTTGGAATGGTATGTAAAGTGA	
YKU70-5-132F	CTCAATTTCATGTATTAGGGATTGC	
YKU70-5-132R-tail	TAGATATGAAGGATTTCAATCGTCTAATATTCCCGTTTTAAAATCAGGC	
YKU70-3-218F	AGACGATTGAAATCCTTCATATCTA	
YKU70-3-218R	ACAACCACATGTTGGAGAATATGC	
PDR1-5-224F	GGCAATAAGAGGCGCTAATTAAAGCT	
PDR1-5-224R-tail	CTTTTATCTATACAAACGTATACGTCTTCCAGTTTCTTGGATTCTTTTCTGTATATTC	
PDR1-3-236F	ACGTATACGTTTGTATAGATAAAAGT	
PDR1-3-236R	ATATCTTTTGTACAATACTAAAATACA	
PDR3-5-214F	TGCCTCCTCTGCCGCTCGGACTTTCC	
PDR3-5-214R-tail	CTGCTTCCCTATTTTCTTTGCGTTTTGCGGTCACGCAATAAGAAAAAATTAATAA	
PDR3-3-233F	AAACGCAAAGAAAATAGGGAAGCAGAG	
PDR3-3-233R	GCTGCATTCCAGGACCTTTTTGAAC	
ERG6-5-217F	CCAATACTTGCTGTTGCCGATAACTTC	
ERG6-5-217R-tail	ATCTTATTGATCTAGTGAATCTTATGCTGCCTACTATATTATTATTTTATTC	
ERG6-3-218F	ATTCACTAGATCAATAAGATTCAAAT	
ERG6-3-218R	TATTCTGATAGAAAATACTGGTCGTTT	
MAG1_dg fw	ATTGGGCCGTATATCTCGCC	Confirmation of disrupted allele by colony PCR
MAG1_dg rv	CGCGATGACCTCTTTGCATG	
MLH1_dg fw	ACTCAGCGTTTGTTTTCGGC	
MLH1_dg rv	TCGGGTCTTTGGTACCGTTG	

MMS2_dg fw	AACATTGCAATGCCGCTCTC
MMS2_dg rv	CAATGCATGAGGTTACCCGC
PSO2_dg fw	ATGGCCTTAATGTGCGTTGC
PSO2_dg rv	GTTTCAACGACAACTCCCGC
RAD10_dg fw	AGTGTCTGTACGGTGGCAAC
RAD10_dg rv	ATCAGCGCCATGGGATAGTG
RAD59_dg fw	AAGAAAGGTTAGCCGACCGG
RAD59_dg rv	CAAACGTATGTGGCCCATGC
YKY70_dg fw	ATCTTGAGATCGGGCGTTCG
YKU70_dg rv	AGGAAGTGGAAACCCTTGGC
PDR1_dg fw	ACGAGAAGAAACGAGCCCTG
PDR1_dg rv	AATAATGGTGGCGAGACGGG
PDR3_dg fw	CTGCCCTCTATGCCCTTGTC
PDR3_dg rv	ATGTCAGACGCGAAGGAGTC
ERG6_dg fw	GTCGTTTACTGCCGTTTCCG
ERG6_dg rv	AAAGCACATGCCGTTTCACC

Table S2. Preparation of repair DNA

Target gene	Primer set for 5'-flanking DNA	Primer set for 3'-flanking DNA	Repair DNA size (bp)
MAG1	MAG1-5-233F/MAG1-5-233R-tail	MAG1-3-222F/MAG1-3-222R	455
MLH1	MLH1-5-206F/MLH1-5-206R-tail	MLH1-3-217F/MLH1-3-217R	423
MMS2	MMS2-5-172F/MMS2-5-172R-tail	MMS2-3-237F/MMS2-3-237R	409
PSO2	PSO2-5-207F/PSO2-5-207R-tail	PSO2-3-175F/PSO2-3-175R	382
RAD10	RAD10-5-182F/RAD10-5-182R-tail	RAD10-3-201F/RAD10-3-201R	383
RAD59	RAD59-5-215F/RAD59-5-215R-tail	RAD59-3-251F/RAD59-3-251R	466
YKU70	YKU70-5-132F/YKU70-5-132R-tail	YKU70-3-218F/YKU70-3-218R	350
PDR1	PDR1-5-224F/PDR1-5-224R-tail	PDR1-3-236F/PDR1-3-236R	460
PDR3	PDR3-5-214F/PDR3-5-214R-tail	PDR3-3-233F/PDR3-3-233R	447
ERG6	ERG6-5-217F/ERG6-5-217R-tail	ERG6-3-218F/ERG6-3-218R	435

Repair DNAs for CRISPR/Cas9-mediated gene disruption were prepared by connecting 5'- and 3'-flanking DNAs by PCR. Target genes, primer sets for preparation of both flanking DNAs for target gene and repair DNA sizes are shown.

Table S3. Primer sets for confirmation of deleted allele by colony PCR

Target gene	Primer set for colony PCR	Wild-type allele (bp)	Deleted allele (bp)
MAG1	MAG1_dg fw/MAG1_dg rv	1723	832
MLH1	MLH1_dg fw/MLH1_dg rv	2945	631
MMS2	MMS2_dg fw_MMS2_dg rv	1313	814
PSO2	PSO2_dg fw/PSO2_dg rv	2847	821
RAD10	RAD10_dg fw/RAD10_dg rv	1269	636
RAD59	RAD59_dg fw/RAD59_dg rv	1392	654
YKU70	YKY70_dg fw/YKY70_dg rv	2330	521
PDR1	PDR1_dg fw/PDR1_dg rv	4031	824
PDR3	PDR3_dg fw/PDR3_dg rv	3774	843
ERG6	ERG6_dg fw/ERG6_dg rv	2007	855

Target genes, primer sets for confirmation of deleted target genes by colony PCR are shown with the expected sizes of colony PCR products from the wild-type and deleted alleles.

Table S4. Statistical analysis by Student's *t*-test for three reporter systems

t-test with luciferase activity (with/without HU)

Pair tested	Reporter system	*p*-value
0 vs. 20 mM	MC	0.23939
0 vs. 50 mM	MC	0.01003
0 vs. 20 mM	SC	0.01946
0 vs. 50 mM	SC	**0.00767**
0 vs. 20 mM	CI	0.01536
0 vs. 50 mM	CI	**0.00049**

Statistical significance of luciferase activity was tested by a two-tailed paired Student's *t*-test between with and without HU in each assay. *P*-values <0.01 are indicated in bold.

t-test with luciferase activity (reporter systems)

Pair tested	HU conc. (mM)	*p*-value
MC vs. SC	0	0.03020
MC vs. CI	0	0.02613
SC vs. CI	0	**0.00333**
MC vs. SC	20	**0.00982**
MC vs. CI	20	**0.00360**
SC vs. CI	20	0.01834
MC vs. SC	50	0.01283
MC vs. CI	50	**0.00595**
SC vs. CI	50	**0.00599**

Statistical significance of luciferase activity was tested by *t*-test between assay systems. *P*-values <0.01 are indicated in bold.

t-test with fold induction (reporter systems)

Pair tested	HU conc. (mM)	*p*-value
MC vs. SC	20	0.04292
MC vs. CI	20	0.01837
SC vs. CI	20	0.03076
MC vs. SC	50	**0.00810**
MC vs. CI	50	**0.00100**
SC vs. CI	50	**0.00208**

Statistical significance of fold induction was tested by *t*-test between assay systems. *P*-values <0.01 are indicated in bold.

Table S5. Statistical analysis by Student's *t*-test with luciferase activities and fold inductions in single-gene deletion mutants treated with four genotoxic chemicals

Strain	MMS conc. (mM)	*p*-value (Luciferase activity)	*p*-value (Fold induction)
mag1Δ	0	**0.00468**	
	0.01	**0.00506**	0.91323
	0.03	**0.00212**	0.90966
	0.1	**0.00462**	0.90328
	0.3	**0.00032**	**0.00565**
	1	**0.00131**	**0.00814**
	10	**0.00746**	**0.00709**
mlh1Δ	0	0.43926	
	0.01	0.05081	0.36160
	0.03	**0.00822**	0.59892
	0.1	0.12363	0.17199
	0.3	0.04356	0.86534
	1	0.36549	0.61371
	10	0.99486	0.55839
mms2Δ	0	0.01969	
	0.01	**0.00468**	**0.00449**
	0.03	**0.00158**	**0.00853**
	0.1	**0.00069**	**0.00360**
	0.3	**0.00064**	**0.00908**
	1	**0.00082**	**0.00091**
	10	**0.00873**	0.25216
pso2Δ	0	0.06790	
	0.01	0.01094	0.37962
	0.03	**0.00352**	0.96342
	0.1	0.02451	0.39530
	0.3	**0.00416**	0.67975
	1	**0.00612**	0.72657
	10	0.75497	0.25940
rad10Δ	0	**0.00297**	
	0.01	**0.00066**	0.27155
	0.03	**0.00047**	0.55885
	0.1	**0.00023**	0.29241
	0.3	**0.00392**	0.72314
	1	**0.00150**	0.61950
	10	0.02815	0.35478
rad59Δ	0	**0.00104**	
	0.01	**0.00012**	0.09356
	0.03	**0.00064**	0.32930
	0.1	**0.00617**	0.04549
	0.3	**0.00115**	0.04833
	1	**0.00085**	**0.00195**
	10	0.02390	0.98851
yku70Δ	0	0.01356	
	0.01	**0.00334**	0.16388
	0.03	**0.00554**	0.65097
	0.1	**0.00586**	0.29693
	0.3	**0.00066**	0.45149
	1	**0.00332**	0.81789
	10	0.10699	0.80863

Strain	CPT conc. (μg/mL)	*p*-value (Luciferase activity)	*p*-value (Fold induction)
mag1Δ	0	**0.00649**	
	5	0.33292	0.59533
	10	0.26506	0.75930
	20	0.40093	0.14982
	40	0.10217	0.30202
	80	0.27750	0.01322
	160	0.87527	0.07196
mlh1Δ	0	0.09184	
	5	0.19564	0.35798
	10	0.13182	0.16142
	20	**0.00320**	0.06286
	40	0.05032	0.39109
	80	0.05974	0.13294
	160	0.03153	0.16013
mms2Δ	0	0.02778	
	5	0.13524	0.58382
	10	0.05175	0.50087
	20	**0.00633**	0.38218
	40	**0.00238**	0.30496
	80	0.10788	0.61409
	160	0.06752	0.55394
pso2Δ	0	0.01713	
	5	0.31368	0.21417
	10	0.02002	0.40849
	20	**0.00919**	0.46865
	40	0.05587	0.07648
	80	0.01190	0.90951
	160	0.02516	0.81383
rad10Δ	0	0.12550	
	5	0.06860	0.08302
	10	0.08166	0.13315
	20	0.67876	0.32014
	40	0.09758	0.08597
	80	0.03421	0.75264
	160	**0.00139**	0.33627
rad59Δ	0	0.04500	
	5	0.04383	0.05895
	10	0.01289	**0.00834**
	20	0.07528	0.09482
	40	0.04938	0.07109
	80	0.17628	0.22487
	160	0.45950	0.74427
yku70Δ	0	0.15032	
	5	0.10540	0.10483
	10	0.73032	0.13320
	20	0.92850	0.06367
	40	0.07028	0.20536
	80	0.25281	0.82006
	160	0.76770	0.05176

Strain	MMC conc. (mM)	*p*-value (Luciferase activity)	*p*-value (Fold induction)
mag1Δ	0	0.03138	
	0.01	0.01293	0.61396
	0.03	0.01646	0.50982
	0.1	**0.00924**	0.46724
	0.3	**0.00236**	0.09187
	1	**0.00799**	0.06263
mlh1Δ	0	**0.00766**	
	0.01	0.01093	0.35881
	0.03	0.03115	0.54085
	0.1	0.01992	0.19908
	0.3	**0.00436**	0.03816
	1	**0.00899**	0.01755
mms2Δ	0	**0.00305**	
	0.01	0.01617	0.73376
	0.03	**0.00535**	0.18589
	0.1	0.01168	0.46170
	0.3	0.01589	0.27589
	1	**0.00363**	0.03549
pso2Δ	0	0.10680	
	0.01	**0.00749**	0.74834
	0.03	**0.00091**	0.21547
	0.1	**0.00096**	0.04977
	0.3	0.01956	0.51348
	1	0.50218	0.50659
rad10Δ	0	**0.00670**	
	0.01	**0.00130**	0.65489
	0.03	**0.00208**	0.59400
	0.1	**0.00175**	0.11649
	0.3	**0.00165**	0.39417
	1	0.14188	0.85591
rad59Δ	0	**0.00532**	
	0.01	**0.00172**	0.89106
	0.03	0.01090	0.07414
	0.1	**0.00376**	0.42867
	0.3	**0.00284**	0.27262
	1	0.09043	0.13336
yku70Δ	0	**0.00841**	
	0.01	**0.00012**	0.33871
	0.03	**0.00129**	0.03572
	0.1	**0.00106**	0.18347
	0.3	**0.00082**	0.30296
	1	**0.00107**	0.06360

Strain	CDDP conc. (mM)	*p*-value (Luciferase activity)	*p*-value (Fold induction)
mag1Δ	0	**0.00179**	
	0.1	0.02996	0.03723
	0.3	0.02334	0.16520
	0.5	**0.00448**	0.01496
	1	0.21036	**0.00326**
mlh1Δ	0	0.01167	
	0.1	**0.00421**	0.68862
	0.3	0.03920	0.95126
	0.5	0.05953	0.27649
	1	0.74702	0.05416
mms2Δ	0	**0.00618**	
	0.1	**0.00333**	0.03627
	0.3	0.02224	0.02865
	0.5	0.11188	0.41637
	1	0.29213	0.01234
pso2Δ	0	0.04554	
	0.1	**0.00373**	0.02424
	0.3	0.29731	0.33405
	0.5	0.08245	0.14625
	1	0.04270	0.20461
rad10Δ	0	**0.00667**	
	0.1	0.01310	0.08664
	0.3	0.01155	0.12880
	0.5	0.03437	0.17146
	1	0.14561	0.31748
rad59Δ	0	0.02437	
	0.1	0.08454	0.20428
	0.3	0.01749	**0.00516**
	0.5	0.25117	0.04704
	1	0.22675	0.26886
yku70Δ	0	0.09617	
	0.1	0.13745	0.39761
	0.3	0.12694	0.38371
	0.5	0.19772	0.04486
	1	0.61753	0.43509

Results from Student's *t*-test (a two-tailed paired) using luciferase activities and fold inductions fromwild-type cells and the correponding gene-deletion mutant treated with indicated concentration of each chemical are shown in each table.
Statistically significant *p*-values (<0.01) are indicated in bold.

Table S6. Statistical analysis by Student's *t*-test of luciferase activities in single-gene deletion mutants in the absence of chemicals

Experiments	*mag1 Δ*	*mlh1 Δ*	*mms2 Δ*	*pso2 Δ*	*rad10 Δ*	*rad59 Δ*	*yku70 Δ*
Exp (MMS)	**0.00468**	0.43926	0.01969	0.06790	**0.00297**	**0.00104**	0.01356
Exp (Phl)	**0.00305**	0.02034	0.02194	0.83373	0.10680	0.05132	0.21940
Exp (MMC)	0.03138	**0.00766**	**0.00305**	0.10680	**0.00670**	**0.00532**	**0.00841**
Exp (CDDP)	**0.00179**	0.01167	**0.00618**	0.04554	**0.00667**	0.02437	0.09617

Results of statistical analyses by *t*-test using luciferase activity in each mutant reporter strain without chemicals are shown in four independent experiments. Chemicals used in each experiment is indicated in parenthesis. Statistically significant *p*-values (<0.01) are indicated in bold.

Table S7. Statistical analysis by *t*-test of luciferase activities and fold inductions in *rad59Δ*-derived double-gene deletion mutants treated with camptothecin (CPT)

Strain	CPT conc. (μg/mL)	*p*- value (Luciferase activity)	*p*- value (Fold induction)
t-test with *rad59Δ*			
mag1Δ/rad59Δ	0	**0.00416**	
	5	0.04789	0.30147
	10	0.02710	0.03922
	20	**0.00455**	0.12904
mlh1Δ/rad59Δ	0	0.02283	
	5	0.04085	**0.00684**
	10	**0.00903**	**0.00296**
	20	0.07232	0.23925
mms2Δ/rad59Δ	0	**0.00735**	
	5	0.33301	0.25991
	10	0.08730	0.02358
	20	0.01964	0.06421
pso2Δ/rad59Δ	0	**0.00037**	
	5	**0.00144**	0.02031
	10	**0.00202**	0.03305
	20	**0.00828**	0.18774
rad10Δ/rad59Δ	0	**0.00558**	
	5	0.12144	0.03113
	10	0.15048	**0.00345**
	20	0.05356	0.34380
yku70Δ/rad59Δ	0	**0.00338**	
	5	0.02540	0.01016
	10	0.21897	**0.00197**
	20	**0.00335**	**0.00983**

Significance of luciferase activities and fold inductions tested by Student's *t*-test between *rad59Δ* cells and the *rad59Δ*-derived double-gene deletion mutant treated with indicated concentration of CPT. Statistically significant *p*-values (<0.01) are indicated in bold.

Table S8. Statistical analysis by Student's *t*-test of luciferase activitives and fold inductions in *mms2Δ*-derived double-gene deletion mutants treated with mitomycin C (MMC)

Strain	MMC conc. (mM)	*p*-value (Luciferase activity)	*p*-value (Fold induction)
t-test with *mms2Δ*			
mag1Δ/mms2Δ	0	0.09567	
	0.01	0.04465	**0.00265**
	0.03	**0.00278**	0.03030
	0.1	**0.00057**	0.03836
	0.3	**0.00133**	0.05030
	1	**0.00649**	0.15101
mlh1Δ/mms2Δ	0	0.03970	
	0.01	0.66332	0.17589
	0.03	**0.00234**	0.02504
	0.1	**0.00179**	0.01036
	0.3	**0.00181**	**0.00949**
	1	**0.00263**	0.01663
pso2Δ/mms2Δ	0	0.02409	
	0.01	0.01501	0.27998
	0.03	**0.00153**	0.26334
	0.1	**0.00020**	0.12309
	0.3	**0.00074**	0.33804
	1	**0.00442**	0.68246
rad10Δ/mms2Δ	0	0.03638	
	0.01	0.01682	0.03549
	0.03	0.07191	0.07579
	0.1	**0.00660**	0.02459
	0.3	0.06235	0.07557
	1	0.01072	0.25762
rad59Δ/mms2Δ	0	0.34493	
	0.01	0.75456	0.49644
	0.03	0.16484	0.17843
	0.1	**0.00768**	**0.00954**
	0.3	0.34164	0.10083
	1	0.33405	0.29109
yku70Δ/mms2Δ	0	0.23091	
	0.01	0.37909	0.07860
	0.03	**0.00179**	0.12714
	0.1	**0.00034**	0.04722
	0.3	0.01467	0.51305
	1	0.01438	0.67500

Statistical significance of luciferase activities and fold inductions tested by Student's *t*-test between *mms2Δ* cells and the *mms2Δ*-derived double-gene deletion mutant treated with indicated concentration of MMC. Statiscally significant *p*-values (<0.01) are indicated in bold.

Table S9. Statistical analysis by *t*-test of luciferase activities and fold inductions in *rad10Δ* and *mms2Δ*-derived double-gene deletion mutants treated with cis-dichlorodiammine platinum (CDDP)

Strain	CDDP conc. (mM)	*p*-value (Luciferase activity)	*p*-value (Fold induction)
t-test with *rad10Δ*			
mag1Δ/rad10Δ	0	0.05946	
	0.1	0.50786	0.12231
	0.3	0.30618	0.34536
	0.5	0.19962	0.69723
mlh1Δ/rad10Δ	0	0.05234	
	0.1	0.68412	0.20491
	0.3	0.34719	0.40522
	0.5	0.10957	0.43936
mms2Δ/rad10Δ	0	0.01264	
	0.1	0.11494	0.50558
	0.3	0.02749	0.26171
	0.5	**0.00058**	0.08112
pso2Δ/rad10Δ	0	**0.00877**	
	0.1	0.95167	0.14000
	0.3	0.69384	0.19342
	0.5	0.15094	0.89112
rad10Δ/rad10Δ	0	0.12838	
	0.1	0.43042	0.17281
	0.3	0.32845	0.45278
	0.5	0.26374	0.59610
yku70Δ/rad10Δ	0	0.06359	
	0.1	0.23545	0.15731
	0.3	0.69298	0.30012
	0.5	0.20719	0.43884

t-test with *mms2Δ*			
mag1Δ/mms2Δ	0	0.05288	
	0.1	**0.00724**	0.16882
	0.3	0.62063	0.36623
	0.5	0.88972	0.13759
mlh1Δ/mms2Δ	0	**0.00221**	
	0.1	**0.00139**	0.04653
	0.3	0.03842	0.77682
	0.5	0.01355	0.03841
pso2Δ/mms2Δ	0	0.36507	
	0.1	0.02711	0.02408
	0.3	0.03533	0.07099
	0.5	0.49046	0.81381
rad10Δ/mms2Δ	0	**0.00007**	

	0.1	**0.00203**	**0.00460**
	0.3	**0.00537**	0.03892
	0.5	**0.00030**	0.62742
rad59Δ/mms2Δ	0	0.15729	
	0.1	0.01049	0.20753
	0.3	0.19125	0.91474
	0.5	0.09160	0.38152
yku70Δ/mms2Δ	0	0.41876	
	0.1	0.01559	0.12472
	0.3	0.09512	0.45537
	0.5	0.03524	0.53728

Statistical significance of luciferase activities and fold inductions tested by Student's t-test between *rad10Δ* and the *rad10Δ*-derived double gene deletion mutant (upper panel), and *mms2Δ* and *mms2Δ*- derived double-gene mutant (lower panel) cells treated with indicated concentration of CDDP. Statistically significant *p* -values (<0.01) are indicated in bold.

Table S10. Statistical analysis by *t*-testof luciferase activities and fold inductions in *pdr1Δ*, *pdr3Δ*, and *erg6Δ*-cells treated with hydroxyurea (HU)

Strain	HU conc. (mM)	*p*-value (Luciferase activity)	*p*-value (Fold induction)
t-test with the wild-type			
pdr1Δ	0	0.01681	
	20	0.02098	0.18931
	50	0.21594	0.05177
pdr3Δ	0	0.04042	
	20	**0.00547**	0.29270
	50	**0.00367**	0.42171
erg6Δ	0	**0.00566**	
	20	0.09884	0.03588
	50	0.04466	0.02593

Statistical significance of luciferase activityies and fold inductions tested by Student's *t*-test between the wild-type cells and indicated gene-deletion strain. Statistically significant *p*-values (<0.01) are indicated in bold.
